# Artificial sensory system based on memristive devices

**DOI:** 10.1002/EXP.20220162

**Published:** 2023-11-20

**Authors:** Ju Young Kwon, Ji Eun Kim, Jong Sung Kim, Suk Yeop Chun, Keunho Soh, Jung Ho Yoon

**Affiliations:** ^1^ Electronic Materials Research Center Korea Institute of Science and Technology (KIST) Seoul Republic of Korea; ^2^ Department of Materials Science and Engineering Korea University Seoul Republic of Korea; ^3^ KU‐KIST Graduate School of Converging Science and Technology Korea University Seoul Republic of Korea

**Keywords:** artificial neuron, artificial receptor, artificial sensory system, artificial synapse, memristor

## Abstract

In the biological nervous system, the integration and cooperation of parallel system of receptors, neurons, and synapses allow efficient detection and processing of intricate and disordered external information. Such systems acquire and process environmental data in real‐time, efficiently handling complex tasks with minimal energy consumption. Memristors can mimic typical biological receptors, neurons, and synapses by implementing key features of neuronal signal‐processing functions such as selective adaption in receptors, leaky integrate‐and‐fire in neurons, and synaptic plasticity in synapses. External stimuli are sensitively detected and filtered by “artificial receptors,” encoded into spike signals via “artificial neurons,” and integrated and stored through “artificial synapses.” The high operational speed, low power consumption, and superior scalability of memristive devices make their integration with high‐performance sensors a promising approach for creating integrated artificial sensory systems. These integrated systems can extract useful data from a large volume of raw data, facilitating real‐time detection and processing of environmental information. This review explores the recent advances in memristor‐based artificial sensory systems. The authors begin with the requirements of artificial sensory elements and then present an in‐depth review of such elements demonstrated by memristive devices. Finally, the major challenges and opportunities in the development of memristor‐based artificial sensory systems are discussed.

## Graphical abstract

The memristor‐based artificial sensory systems composed of integrated and cooperative parallel networks of artificial sensory receptors, neurons, and synapses are reviewed. Specific mechanisms and critical functions required by the memristive device are reviewed. The reported studies that functionally implement artificial sensory systems with memristive devices are introduced, discussing the limitations and future research directions in developing memristor‐based artificial sensory systems.

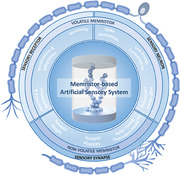

## 1 INTRODUCTION

The escalating demand for automated systems in various fields such as supply chains,^[^ ^1^ ^]^ manufacturing,^[^ ^2^ ^]^ robotics,^[^ ^3^ ^]^ and unmanned vehicles^[^ ^4^ ^]^ has driven advancements in artificial intelligence (AI) technologies, which promise substantial improvements in efficiency and autonomy across various industries. These technologies depend on sensory systems composed of sensors and computational networks to sense the surroundings and acquire real‐time environmental information.^[^ ^5^, ^6^, ^7^, ^8^ ^]^ Traditional complementary metal‐oxide semiconductor (CMOS)‐based sensory systems have promising applications in intelligent recognition and control, such as image classification, language processing, speech recognition, and decision‐making tasks.^[^ ^9^, ^10^ ^]^ However, because these systems based on the von Neumann architecture have distinct memory and processor units, they involve substantial data movement between these units.^[^ ^11^, ^12^, ^13^ ^]^ This data movement results in significant latency, known as the “von Neumann bottleneck,” which limits their computing speed and energy efficiency, ultimately degrading the performance of AI applications.

Biological sensory systems detect, interpret, and store external information in a data‐parallel and integrated manner.^[^ ^14^, ^15^ ^]^ Unlike the conventional electronic sensing systems, human cognitive and memory systems have preprocessing functions in addition to the basic functions of detecting and collecting information from the environment.^[^ ^16^, ^17^ ^]^ Instead of directly delivering all the received external signals to the brain, preprocessing greatly reduces the time and power burden of processing large amounts of analog environmental signals.^[^ ^18^ ^]^ For example, the human sensory system performs a complex function known as adaptation,^[^ ^19^, ^20^ ^]^ such as tactile adaptation. When we wear clothes, we initially recognize the sensation of the fabric on our skin; however, over time, we no longer notice this sensation. Our sensory nerve cells do not continue to send signals to the brain about the constant pressure on our skin.^[^ ^3^, ^21^ ^]^ Instead, they adapt and stop firing, saving energy, and allowing us to process other related stimuli. This ability allows us to explore and interact with complex environments without being overwhelmed by irrelevant, redundant, or unchanging sensory information.^[^ ^22^, ^23^, ^24^ ^]^ In other words, our neural system efficiently manages the computational load and power consumption while focusing resources on new and potentially important stimuli.

These sensory systems process data through interconnected networks of neurons and synapses, based on a distributed computing paradigm, exhibiting superior fault tolerance, power efficiency, and adaptability.^[^ ^25^, ^26^ ^]^ In biological sensory systems, the detection, transmission, and processing of information rely on distributed and parallel architectures of receptors, neurons, and synapses.^[^ ^21^, ^27^, ^28^, ^29^ ^]^ Outer stimuli with environmental information are detected by receptors, encoded by neurons into action potentials in the form of spikes,^[^ ^18^, ^30^ ^]^ and transmitted across the synapses. The signals are then combined in a synergistical way to process the detected information with learning and inference.^[^ ^3^, ^16^, ^31^ ^]^ Inspired by the functions and architectures of these biological systems, researchers have developed neuromorphic computing technologies to address the technical challenges of conventional CMOS‐based sensory systems. Studies have already been conducted to mimic the behaviour of biological neurons and synapses using conventional CMOS technology. However, these conventional CMOS‐based sensory systems require many unit transistors to mimic neurons. It can make the system complex, leading to inefficiencies in the area and energy consumption. For example, Loihi, Intel's neuromorphic research chip designed to mimic how brains work and promise substantial performance and energy efficiency gains for specific AI workloads, requires many transistors (2 × 10^9^). While a biological synapse can show a vast range of synaptic weights, representing each state in silicon requires multiple transistors per synapse, significantly increasing the transistor count. The power consumption in such structures is high due to switching costs, leakage current, communication overhead, and integration challenges. Every time a transistor switches, there's an associated energy cost. The constant operation and updating of synaptic weights mean a lot of switching, leading to high power consumption. Sending signals between transistors consumes energy, especially when the architecture is not optimized for such communication. They also pack many transistors into a small area, resulting in heating and associated power and efficiency challenges. Furthermore, ADC converting is required because of digital‐based processing, and it cannot be applied to flexible electronics (e.g. substrates) when integrated with other sensors. Therefore, complexity, power consumption, and area inefficiencies in conventional CMOS‐based sensory systems are some of the challenges that researchers continue to address. Whereas, in neuromorphic computing systems, sensory information is integrated, processed, and memorized—serving as a critical factor in decision‐making, cognition, or learning/memory tasks. These system can execute multiple tasks in highly parallel contexts, with a low power consumption of approximately 1−100 fJ per synaptic event.^[^ ^32^ ^]^

Although conventional transistor‐based CMOS technologies have been developed for neuromorphic computing, they are inefficient in terms of scalability and energy usage. Memristive devices have emerged as highly advantageous and critical hardware technologies for implementing artificial sensory systems, primarily owing to their distinct properties and inherent capabilities to mimic those of biological sensory systems.^[^ ^3^, ^33^, ^34^, ^35^, ^36^ ^]^ At the fundamental level, memristors have a relatively simple two‐terminal structure that can change its resistance state based on the applied voltage and current history. Depending on the degree of retainment of the resistance state, memristors are classified as volatile^[^ ^37^, ^38^, ^39^, ^40^ ^]^ and non‐volatile memristors.^[^ ^41^, ^42^, ^43^, ^44^ ^]^ In the case of a volatile memristor, the resistance state temporarily changes from a high resistance state (HRS) to a low resistance state (LRS), when a voltage above a threshold value is applied. It recovers from the LRS to HRS after the removal of the applied voltage. This temporary threshold‐switching characteristic of the volatile memristor is used to implement biological receptors and neurons, which also have the nature of threshold response.^[^ ^45^, ^46^, ^47^, ^48^, ^49^, ^50^ ^]^ On the contrary, non‐volatile memristors can maintain the resistance change corresponding to an applied voltage, for a long time, making them excellent candidates for mimicking biological synapses.^[^ ^51^, ^52^, ^53^, ^54^, ^55^ ^]^ This characteristic, combined with their scalability and power consumption that is lower than that of the traditional CMOS devices, allows them to effectively mimic the functions of biological receptors, neurons, and synapses. The ability of memristive devices to regulate their dynamics between volatile and non‐volatile states, according to the device structure and material, provides significant advantages by expanding their applicability and scope. They can be fabricated from a wide range of materials including oxides, chalcogenides,^[^ ^56^, ^57^, ^58^ ^]^ and organics,^[^ ^59^, ^60^, ^61^, ^62^, ^63^ ^]^ and the choice of material can significantly affect the device performance characteristics such as switching speed, endurance, and retention properties, offering numerous options for customization according to specific requirements. Moreover, the memristor can be designed with various structures such as planar crossbar arrays and three‐dimensional vertical configurations, providing unique advantages in terms of the integration density, power efficiency, and switching properties.^[^ ^64^, ^65^, ^66^, ^67^, ^68^ ^]^ Most importantly, memristors can emulate various biological mechanisms involved in the sensing, processing, and storage of information. This ability allows them to effectively mimic the functionality of artificial sensory systems including artificial sensory receptors, neurons, and synapses, making them essential for the development of advanced neuromorphic systems.

**FIGURE 1:**
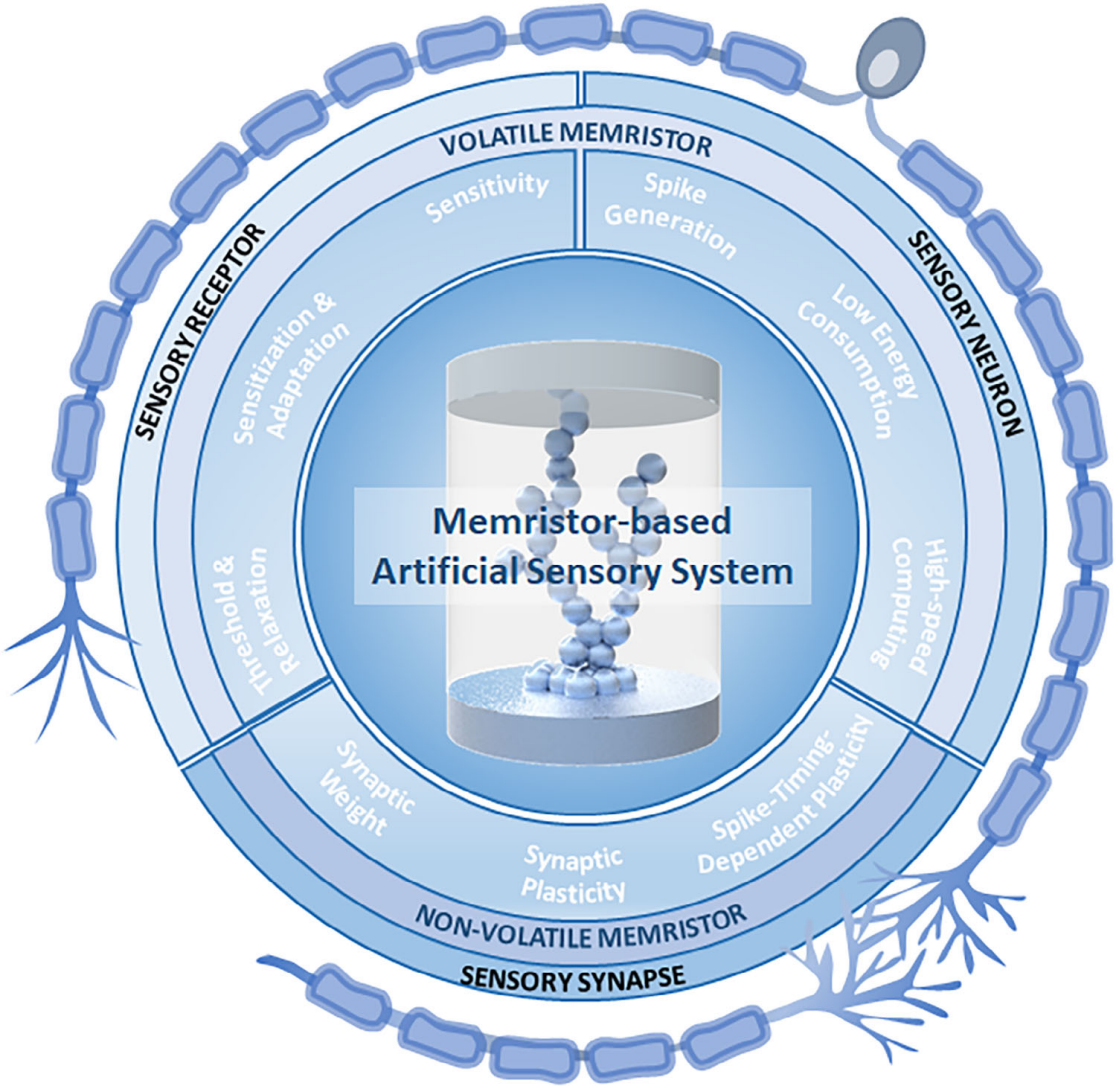
Schematic illustration of the memristor‐based artificial sensory system composed of integrated and cooperative parallel networks of artificial sensory receptors, neurons, and synapses.

This review explores the current state‐of‐the‐art memristor‐based artificial sensory system comprising sensory receptor, neuron, and synapse systems. As depicted in Figure 1, we present the requirements and critical characteristics necessary to implement each system element. We describe the specific mechanisms and functionalities applied in the process of implementing with memristors the roles of sensory receptors, sensory neurons, and sensory synapses. The sensory receptors detect stimuli from the external environment and produce continuous receptor potentials in accordance with their specific receptor characteristics. After that, sensory neurons generate spike‐based action potentials according to stimuli. Sensory synapses memorize and store the intensity of the stimuli through changes in synaptic weight. The studies that functionally implement artificial sensory systems with memristive devices are detailed in this review. So far, most memristor‐based artificial sensory system studies have focused mainly on the fabrication and improvement of individual sensory elements, and integrated artificial sensory systems with all functional elements have yet to be explored, and many challenges exist. Therefore, in this review, we provide a device understanding of each sensory element and present the challenges to be solved for practical application. As biological sensory systems operate via the integration and cooperation of parallel networks of receptors, neurons, and synapses, this review focuses on both the studies that have developed each artificial element individually and those that consider integrated approaches. Finally, we discuss the ongoing challenges and prospects in the development of memristor‐based artificial sensory systems.

## 2 BIO‐INSPIRED ARTIFICIAL SENSORY ELEMENTS

### 2.1 Artificial sensory receptors

Biological sensory receptors play a crucial role in sensory transduction, converting external stimuli into electrical signals that the biological nervous system can interpret. These sensory receptors are specialized elements that detect changes in the outer environment and trigger impulses (receptor potentials) within the sensory nervous system, acting as a critical intersection between the outer information and internal nervous system in all vertebrates.^[^ ^69^, ^70^, ^71^ ^]^ Located ubiquitously throughout the body, these receptors allow organisms to sense, perceive, and interact with their surroundings. They are activated when external stimuli exceed a specific threshold, and their response to prolonged stimulation is characterized by adaptation or maladaptation.^[^ ^72^, ^73^ ^]^ Adaptability is crucial for organisms to filter out repetitive and innocuous information and recognize potentially damaging stimuli.

Sensory receptors are primarily classified based on their adaptation rate into three categories: rapid, slow, and non‐adapting receptors.^[^ ^74^ ^]^ Rapid and slow adapting receptors are activated by innoxious stimuli that surpass the threshold value, and they adapt by lowering their sensitivity, allowing organisms to eliminate extraneous and recurring information.^[^ ^75^, ^76^ ^]^ On the other hand, non‐adapting receptors, also known as nociceptors, are activated by high‐threshold stimuli that are potentially harmful to tissues, and they do not adapt to these noxious stimuli, leading to conscious awareness of pain.^[^ ^77^, ^78^, ^79^ ^]^ Thus, an organism's response to different stimuli—innocuous or noxious—plays a crucial role in its survival.

**FIGURE 2:**
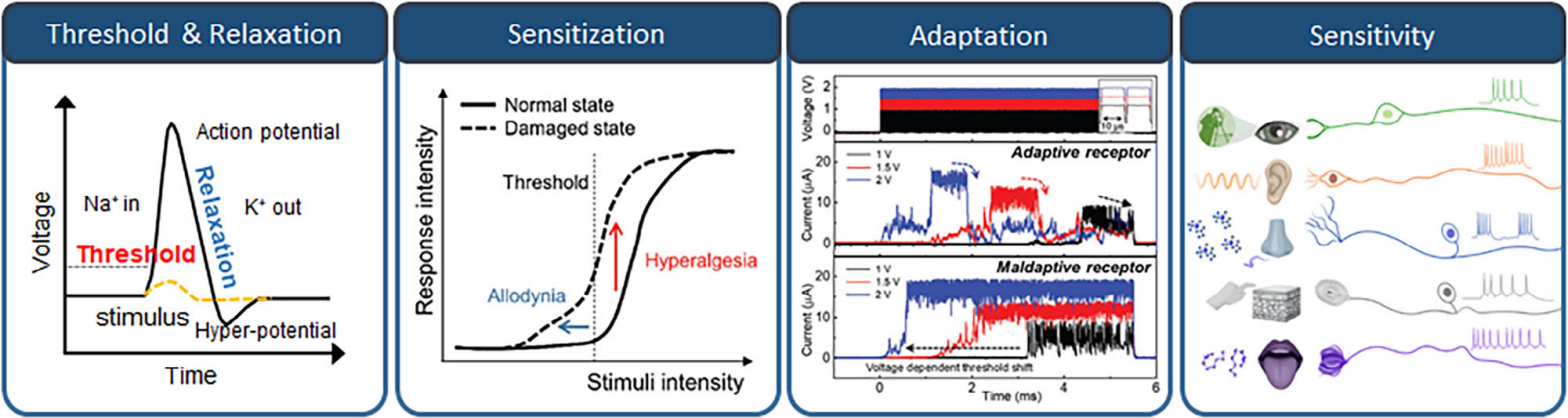
Features and performances required to implement artificial sensory receptors, which involve “Threshold and Relaxation,” “Sensitization”, “Adaptation,” and “Sensitivity” to external stimuli. (Sensitization) Reproduced with permission.^[^ ^79^ ^]^ Copyright 2018, WILEY‐VCH Verlag GmbH & Co. KGaA, Weinheim. Reproduced under the terms of the Creative Commons CC BY license.^[^ ^74^ ^]^ Copyright 2021, The Authors, published by Wiley‐VCH GmbH. Reproduced under the terms of the Creative Commons CC BY license.^[^ ^86^ ^]^ Copyright 2021, The Authors, published by Springer Nature.

Inspired by these biological systems, bio‐inspired electronics aim to imitate the sensory transmission process of biological receptors. This involves converting an outer stimulus into an electrical signal, transmitting this signal in a manner similar to that employed by biological neurons, and emulating synaptic plasticity.^[^ ^3^, ^80^, ^81^, ^82^ ^]^ This section describes the roles and functions of sensory receptors in sensory nervous systems and the functional elements required to implement them. As depicted in Figure 2, the functional elements of artificial sensory receptors performing “threshold and relaxation,” “sensitization and adaptation,” and “sensitivity” to external stimuli, will be discussed.

#### 2.1.1 Threshold and relaxation

Understanding the principles of the threshold and relaxation functions in biological receptors is important to develop efficient artificial sensory receptors. Biological receptors located at the outermost edges of our sensory nerves are responsible for responding to external stimuli. They generate initial biochemical signals based on the intensity, duration, and frequency of the stimuli.^[^ ^34^ ^]^ A critical feature of these receptors is their threshold behaviour. They remain unresponsive to stimuli that fall below a certain threshold value, but exhibit a strong response to stimuli that exceed this value. This ability to discriminate between different levels of stimuli is a crucial aspect of sensory perception. Following activation by an over‐threshold stimulus, the receptors undergo a relaxation period during which they slowly turn off. Interestingly, during this relaxation phase, the stimulus required to re‐ignite the receptors is below the initial threshold value, because the receptors remain partially activated after the initial response; therefore, less stimulus is needed for full activation. These threshold and relaxation behaviours have important implications for the survival and well‐being of the organism. They enable the body to avoid or mitigate damaging and continuous stimuli—elements perceived as dangerous. By understanding and emulating these behaviours, we can design artificial sensory receptors that not only mimic biological functions but also offer the ability to tune responsiveness based on specific needs or environments.

#### 2.1.2 Sensitization and adaptation

Another critical aspect of biological sensory receptors that should be considered in the design of artificial sensory receptors is the phenomenon of sensitization and adaptation. “Sensitization” is a hypersensitive reaction of a damaged nociceptor and “adaptation” is a phenomenon in which the output signal is reduced when repeated innoxious stimuli arrive at a normal sensory receptor. Sensitization is particularly relevant when studying nociceptors, which are responsible for detecting potentially harmful stimuli. Under normal conditions, nociceptors have a threshold below which they do not respond. However, when these receptors encounter strong stimuli that can cause damage, they can enter an abnormal state, which alters their response behaviour. This state is characterized by two main phenomena: allodynia and hyperalgesia.^[^ ^83^ ^]^ Allodynia refers to a condition in which responses are triggered even by stimuli that fall below the usual threshold. Meanwhile, hyperalgesia describes an excessive response to stimuli that exceed the threshold, generating signals stronger than that generated normally. These changes in responsiveness in the abnormal state are protective mechanisms of the body. They enable the body to respond immediately to harmful stimuli, helping to prevent further damage. An example for this is the heightened sensitivity to heat, which is often observed in burnt skin cells.^[^ ^84^ ^]^

Adaptation behaviour is also very crucial in the human sensory system. Adaptive receptors react to innoxious external stimuli that exceed the threshold value and show adaptive behaviours by decreasing the receptor sensitivity. With this feature, receptors need not process the repetitive innoxious data that can decrease efficiency. By mimicking these complex responses of biological systems, artificial receptors that are sensitive and adaptive to the complex dynamics of the environment can be developed. The implementation of such complex functionalities of nociceptors and normal sensory receptors using traditional sensors and CMOS‐based circuits can be extremely challenging. In this context, memristive devices offer a promising alternative, as they can mimic the information sensing and generation roles of biological receptors and their more conventional roles in information storage and processing.

#### 2.1.3 Sensitivity

Sensitivity refers to the ability of sensory receptors to react to changes in external stimuli. The functioning of sensory receptors—biological or artificial—is inherently related to their sensitivity to external stimuli. This sensitivity means how effectively these receptors detect, respond to, and transmit information on external stimuli to the nervous system or corresponding artificial system. Therefore, the development of artificial sensory receptors should consider and incorporate sensitivity as a functional element. In this context, the more sensitive a receptor is, the better it can identify subtle variations in the environment. This ability can be crucial in various applications, from detecting potential threats in safety systems to discerning nuanced sensory information in robotics. Biological receptors exhibit an incredible range of sensitivities, allowing organisms to perceive their environment with great detail and adapt accordingly. For instance, human eyes can detect a single photon, the smallest unit of light.^[^ ^85^ ^]^ Similarly, the skin, which is the largest organ, is densely packed with different types of sensory receptors, enabling organisms to feel even the slightest changes in pressure, temperature, or vibration.

Mimicking this level of sensitivity in artificial sensory receptors poses significant challenges. Nevertheless, it offers enormous potential for improving the performance and versatility of artificial systems. Advances in materials science, nanotechnology, and signal processing, among other fields, are helping to increase the sensitivity of artificial sensory receptors. A key focus in enhancing sensitivity is ensuring that artificial receptors can detect a wide range of stimulus intensities. They should be capable of discerning weak signals but also robust enough not to be overwhelmed by strong stimuli. They should also be resilient, maintaining their sensitivity over time and under varying environmental conditions.

### 2.2 Artificial sensory neurons

Biological sensory systems exhibit efficient data processing by utilizing spike‐based signal transmission to significantly reduce the energy consumption. An artificial sensory system that mimics these capabilities using hardware‐based artificial neural networks with neuromorphic architectures is a promising solution for low‐power sensory processing.^[^ ^87^, ^88^, ^89^ ^]^ To interface with a spiking neural network (SNN), external signals must be converted into spike forms, which cannot be achieved using the conventional passive sensors. This necessity has spurred the development of artificial neurons. Much like their biological counterparts, artificial neurons generate spike signals as output, only when the input transmitted from the synapses surpass a certain threshold. This threshold function is crucial for judging the sufficiency of the signals collected from the synapses. Most artificial neurons receive the current input from the preceding synapses and generate voltage output to the subsequent synapses, in spiking or “firing” form.^[^ ^90^ ^]^

**FIGURE 3:**
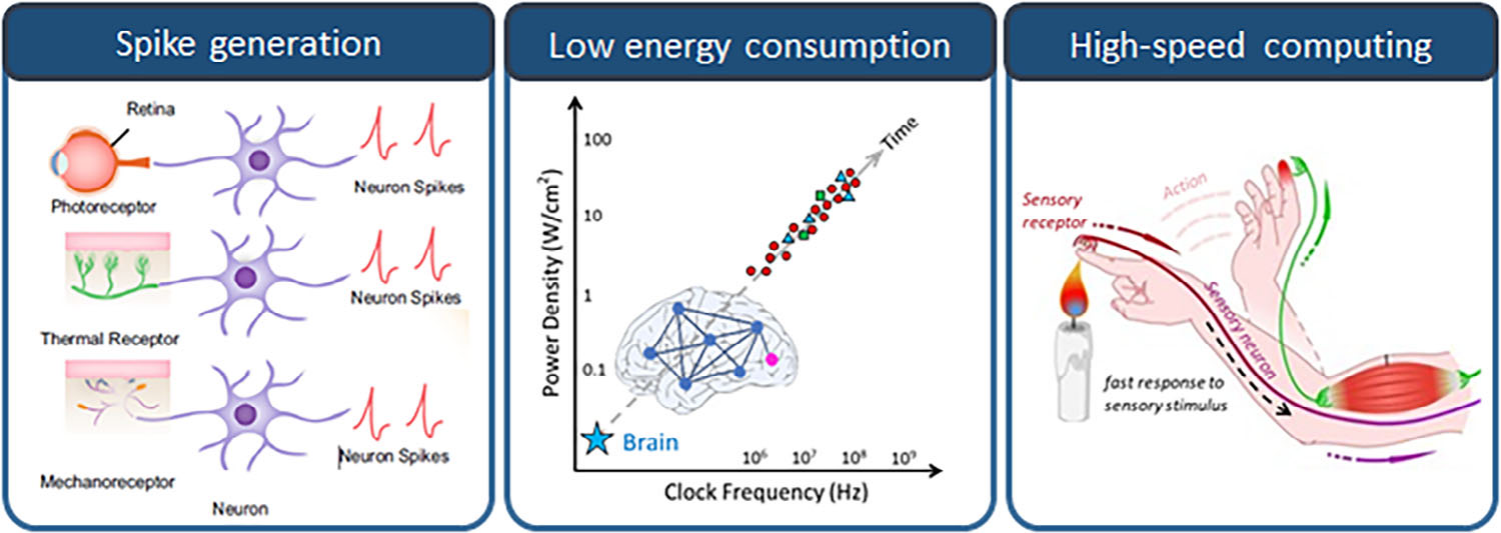
Features and performances required to implement artificial sensory neurons, which include “Spike generation,” “Low energy consumption,” and “High‐speed computing.” (Left, spike generation) Reproduced under the terms of the Creative Commons CC BY license.^[^ ^160^ ^]^ Copyright 2022, The Authors, published by Springer Nature.

Artificial sensory neurons, capable of simultaneous neural processing and sensing, can convert continuous analog signals from the external environment into discrete electrical spike signals, without requiring additional hardware or conversion circuits. Such “in‐sensor computing” enables the direct transformation of continuous analog signals from the external environment into discontinuous spike signals within the sensor.^[^ ^91^ ^]^ This eliminates the need for conversion circuits like analog‐to‐digital converters or hardware components typically used in von Neumann architectures. This can substantially reduce both the cost of the hardware and the energy consumed by the sensory system, making these devices particularly well‐suited for applications in mobile and Internet of Things sensors.^[^ ^9^ ^]^ This section presents the critical functions of artificial sensory neurons in sensory systems and the essential functional requirements for their implementation, which are depicted in Figure 3.

#### 2.2.1 Spike generation

The spike output is a key functionality of artificial sensory neurons. Similar to biological neurons, artificial neurons must generate a “spike” (action potential) when the input surpasses a certain threshold. This spike generation allows to encode sensory information in a binary language that the neural network can understand. The spiking mechanism, which mimics the essential communication method found in biological neural networks, has evolved over millions of years and has become incredibly efficient at processing complex sensory information. Spike generation is typically achieved through the use of artificial neuron devices with threshold‐switching characteristics, which allow spike generation when the input signal surpasses a certain threshold.^[^ ^92^ ^]^ The exact threshold can be adjusted based on the needs of the system, allowing flexible and adaptable sensory processing. By encoding information in the form of spikes, artificial neurons can effectively reduce the amount of data that needs to be processed by the subsequent layers of the network, improving the efficiency and reducing the energy consumption. It also allows for real‐time, dynamic processing of sensory information, which is critical for applications such as robotic sensory systems.^[^ ^93^ ^]^

#### 2.2.2 Low energy consumption

Unlike the conventional computing systems that consume significant amounts of power, neuromorphic computing systems are designed to mimic the energy efficiency of biological brains. By implementing the structure of the human brain and its computational operations in hardware, information processing and storage can be achieved simultaneously and parallelly, using the same component, enabling efficient operation at high speed and low power. As neuromorphic computing hardware is composed of artificial neurons and synaptic devices, many studies have already been conducted to simulate the behaviour of biological neurons using CMOS‐based integrated circuits based on the established silicon semiconductor process technology. However, they are inefficient in terms of the area and energy consumption because a considerable number of unit transistors are required to simulate neurons, which complicates the system.^[^ ^94^, ^95^ ^]^ Moreover, considering that the operating voltage of biological neurons is significantly lower than 100 mV,^[^ ^96^ ^]^ the development of artificial neuron devices with low operating voltage levels is important for neuromorphic hardware implementation.

Therefore, research on memristor‐based artificial neuron devices is being conducted to overcome these limitations and implement energy‐efficient neuromorphic computing hardware. Owing to their threshold‐switching characteristics, these devices can operate at lower power levels by enabling to control the resistance state transition precisely and efficiently. Since volatile memristors only switch states when a specific threshold voltage is applied, they can achieve state transitions with minimal energy input. This efficiency is particularly beneficial for applications that require frequent state changes while aiming to conserve power. More importantly, these devices can generate spike signal outputs and operate under the principles of spike‐based computing, a method that the brain uses to process information with remarkable energy efficiency. Unlike traditional computing systems that process data in a clock‐driven manner (constantly processing at fixed intervals), neuromorphic systems process data based on events or spikes. This event‐driven process means that computations are only done when necessary, mimicking the behaviour of biological neurons that fire only when a certain activation threshold is reached. In other words, the event‐driven nature of neuron‐based neuromorphic or artificial sensory systems offers a fundamentally different computing paradigm, which inherently supports energy‐efficient operations. Additionally, these devices can generate spike signal outputs and operate under the principles of spike‐based computing, a method that the brain uses to process information, with remarkable energy efficiency.^[^ ^97^, ^98^ ^]^ In this regard, memristor‐based artificial neuron devices present the possibility of developing power‐efficient neuromorphic systems that can perform complex computations with less energy. As the implementation of such power‐efficient computing systems requires careful consideration of several factors such as device design, material selection, and integration of devices into larger systems, we will examine the current state of the technology, remaining challenges, and potential solutions to overcome these challenges to realize power‐efficient spike‐based neuromorphic computing.

#### 2.2.3 High‐speed computing

Speed is a critical aspect of sensory processing, especially, when dealing with real‐time data in diverse environments. Artificial sensory neurons must quickly respond to sensory stimuli to ensure rapid signal processing and response to changing surroundings. The speed of computation in artificial neurons is influenced mainly by the switching speed, which essentially determines how fast an artificial neuron can “fire,” or generate spikes in response to stimuli.^[^ ^99^ ^]^ Higher switching speeds imply that the artificial neuron can process more sensory data over a given time, leading to a faster and more efficient sensory system. This aspect is particularly important when these systems are utilized in applications such as autonomous vehicles, robotics, or real‐time monitoring systems, where even minor delays in processing sensory information can have significant consequences.^[^ ^100^ ^]^ When performing high‐speed calculations, researchers face challenges in managing energy efficiency, maintaining integrity of processed information, and ensuring stability of artificial neurons.^[^ ^101^ ^]^ Therefore, research on artificial sensory neurons aims to design efficient and responsive neuron devices to solve these challenges. Memristive devices with superior switching speeds, low‐power operation, and high integration capabilities are emerging as alternatives.^[^ ^102^ ^]^

### 2.3 Artificial sensory synapses

**FIGURE 4:**
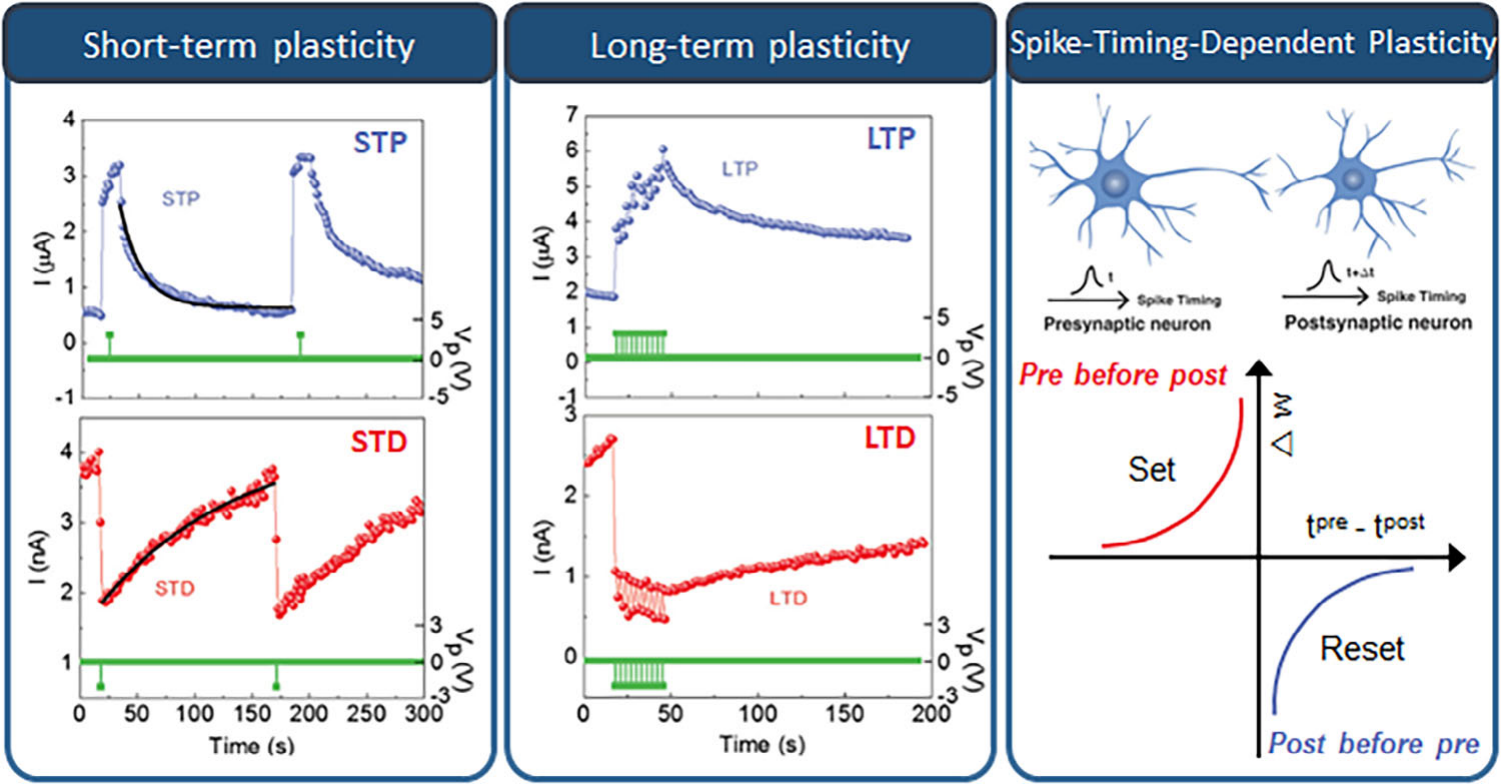
Features and performances required to implement the artificial sensory synapse, which involve “Short‐term plasticity” (Short‐term potentiation and depression (STP and STD)), “Long‐term plasticity” (Long‐term potentiation and depression (LTP and LTD), and “Spike‐timing–dependent plasticity”. (Left, Middle) Reproduced with permission.^[^ ^126^ ^]^ Copyright 2019, WILEY‐VCH Verlag GmbH & Co. KGaA, Weinheim. (Right) Reproduced under the terms of the Creative Commons CC BY license.^[^ ^127^ ^]^ Copyright 2022, The Authors, published by Wiley‐VCH GmbH.

Connecting one neuron's axon to another neuron's dendrite, known as a synapse, plays a critical role in neural communication. At the synapse, the input signal from the axon of the pre‐neuron is modulated based on the strength of the synaptic connection. This strength can be potentiated or depressed in response to input stimuli. It is widely recognized as the fundamental mechanism underlying memory and learning functions within the human brain.^[^ ^3^ ^]^ In this section, as depicted in Figure 4, we will explore the features and performances required to implement an artificial sensory synapse.

#### 2.3.1 Synaptic weight

Synaptic weight is a parameter that quantifies the strength of the connection between two neurons and determines the effect of a neural activity on another in a neural network, representing the effectiveness or influence of the connection.^[^ ^103^, ^104^, ^105^ ^]^ It can be quantified as a positive or negative value, indicating the excitatory or inhibitory nature of the synapse. The value of the synaptic weight is adjusted during the learning process of a neural network to optimize its performance on specific tasks or patterns. Synaptic plasticity is the ability of synaptic connections to change and adapt over time.^[^ ^106^, ^107^, ^108^ ^]^ It is the property that allows synapses to undergo modifications to their strength, efficiency, and structure in response to activity and experience. Synaptic plasticity plays a crucial role in learning, memory formation, and information processing in the brain, allowing the synaptic weight to be modified by either increasing or decreasing its value. This will be explained in the following subsection.

#### 2.3.2 Short‐term plasticity and long‐term plasticity

Short‐term plasticity (STP) refers to the transient changes in synaptic strength, which occur over a short period—typically within seconds to minutes, as shown in Figure 4.^[^ ^109^, ^110^ ^]^ STP is achieved through the temporal enhancement of a synaptic connection, which then quickly decays to its initial state. There are two main forms of STP: short‐term potentiation and short‐term depression. Short‐term potentiation refers to an increase in the synaptic strength, resulting in a stronger postsynaptic response, whereas short‐term depression refers to a decrease in the synaptic strength and a weaker postsynaptic response. STP is a dynamic process that allows synapses to adapt to varying patterns of neural activity. It modulates the strength and timing of synaptic transmission, which can have important implications in information processing in neural circuits. For example, STP, which is commonly observed in synapses engaged in rapid and sustained signalling like sensory processing or motor control, is crucial for filtering out irrelevant information.

In contrast to STP, long‐term plasticity (LTP) involves enduring modifications in synaptic strength and is crucial for neural‐network function and learning.^[^ ^111^, ^112^, ^113^ ^]^ Long‐term potentiation and long‐term depression play vital roles in synaptic plasticity. Long‐term potentiation strengthens synapses through molecular and structural changes, enhancing responsiveness, whereas long‐term depression weakens synapses, optimizing information processing efficiency by eliminating less active or less relevant connections (Figure 4). LTP is a crucial mechanism for cellular and molecular learning and memory formation. The dynamic process of synaptic plasticity, involving both long‐term potentiation and long‐term depression, allows the brain to learn new skills, form memories, and adapt its neural circuits. The brain can encode and store information based on experiences and environmental stimuli by modifying synaptic connections. These modifications enable the brain to retrieve and recall memories, associate related information, and adapt cognitive processes to changing circumstances.

#### 2.3.3 Spike‐timing‐dependent plasticity

Another important characteristic of the synapse is the spike‐timing–dependent plasticity (STDP), a time‐dependent specialized feature of Hebbian learning^[^ ^114^, ^115^, ^116^, ^117^ ^]^ (Figure 4). Hebbian learning is a theory in neuroscience, which describes a fundamental principle of synaptic plasticity—the ability of synapses to change their strengths based on neuronal activity patterns. STDP demonstrates that, when two neurons are repeatedly and persistently activated together, the connection between them is strengthened. When a presynaptic neuron consistently activates a postsynaptic neuron, the synapse between them becomes stronger. Hebbian learning is based on the idea that the brain learns by forming associations between co‐occurring neural activities. A common goal of STDP learning is increasing the efficiency of synapses when two events occur in the expected order, and decreasing it when they do not. The STDP rule defines the change in synaptic weight as a specified function, *f*_STDP_, of the difference between the firing times of pre‐(*t*_pre_) and post‐synaptic (*t*_post_) spikes. Specifically, the synaptic weight is increased when a presynaptic spike comes before a postsynaptic spike by a short interval. In contrast, as shown in Figure 4, if the postsynaptic spike precedes the presynaptic spike, the synaptic weight diminishes, while no change occurs when the time interval between the pre‐ and post‐synaptic spikes is too large.

A few additional types of STDP have been identified, namely, anti‐Hebbian STDP,^[^ ^117^, ^118^ ^]^ symmetric STDP,^[^ ^119^, ^120^, ^121^ ^]^ and STDP observed across various synapse types.^[^ ^122^ ^]^ Anti‐Hebbian STDP is a modified version of STDP, which functions in a manner contrary to that of Hebbian learning. In anti‐Hebbian STDP, the synaptic weight is reduced when the presynaptic spike precedes the postsynaptic spike. This type of plasticity is often linked to synaptic pruning, which selectively eliminates weaker connections to refine neural circuits and optimize information processing. Thus, although the underlying assumptions remain consistent, the distinguishing factor lies in the chronological order of the synaptic events.^[^ ^123^, ^124^, ^125^ ^]^

## 3 MEMRISTOR‐BASED ARTIFICIAL SENSORY SYSTEM

### 3.1 Implementation of artificial receptor with memristive devices

**FIGURE 5:**
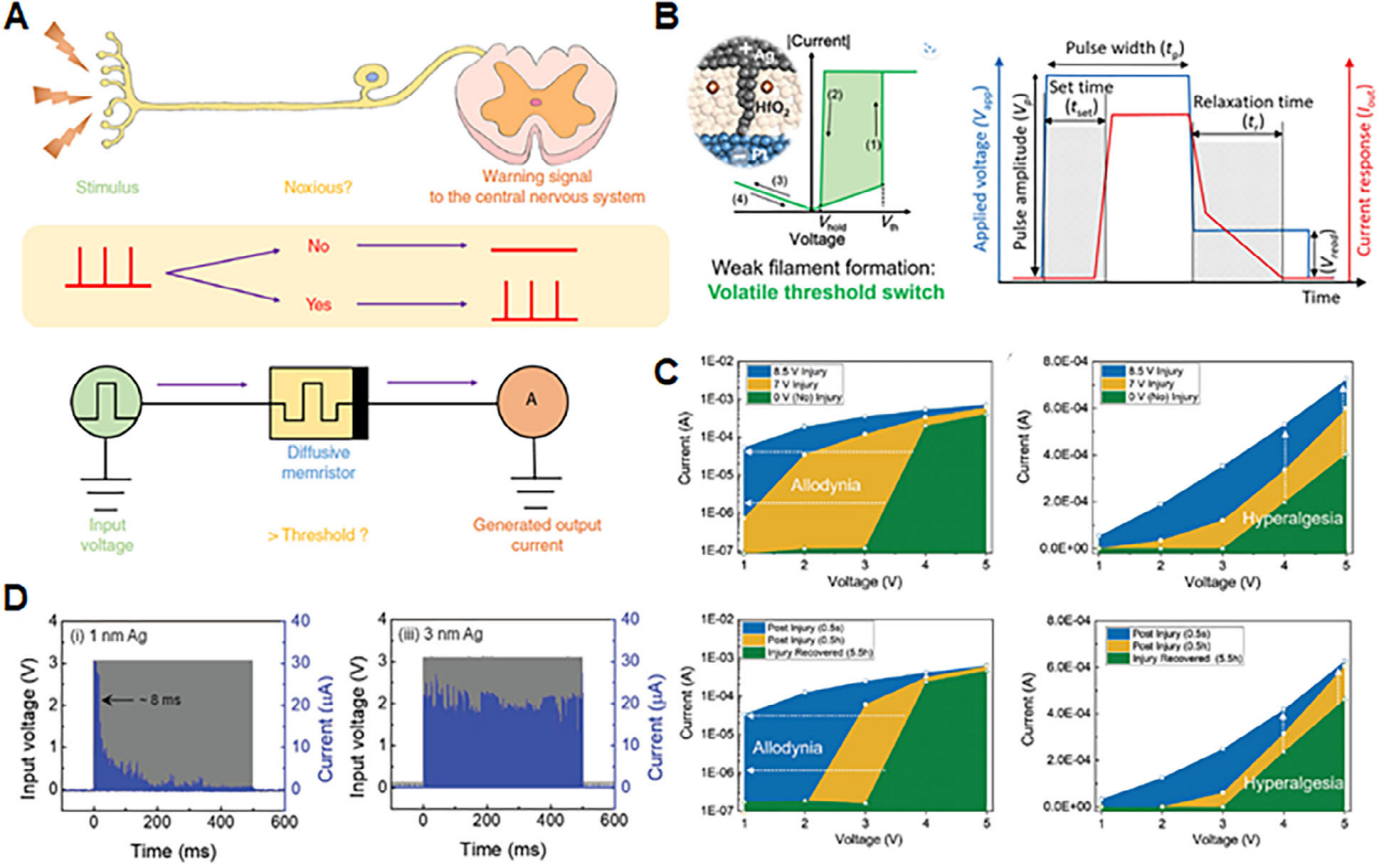
(A) Working principle of nociceptor and its biological counterpart. Artificial nociceptor circuit consisting of a diffusive memristor. Reproduced under the terms of the Creative Commons CC BY license.^[^ ^128^ ^]^ Copyright 2018, The Authors, published by Springer Nature. (B) Schematic graph defining the *t*_set_ and *t*_r_ from the temporal response measurement. The blue line represents the applied voltage (*V*_app_) and the red line represents the output current response (*I*_out_). Reproduced under the terms of the Creative Commons CC BY license.^[^ ^134^ ^]^ Copyright 2021, The Authors, published by Wiley‐VCH GmbH. (C) Demonstration of sensitization features according to different injury pulses and time intervals. Reproduced under the terms of the Creative Commons CC BY license.^[^ ^130^ ^]^ Copyright 2022, The Authors, published by Wiley‐VCH GmbH. (D) The adaptation rate of different Ag amount memristors demonstrated by pulse response (left: small amount, right: large amount of Ag‐based memristors). Reproduced under the terms of the Creative Commons CC BY license.^[^ ^74^ ^]^ Copyright 2021, The Authors, published by Wiley‐VCH GmbH.

Memristors have effectively shown the ability to emulate biological neuronal actions and synaptic functions including the transition from STP to LTP, STDP, and spike‐rate–dependent plasticity. Although the implementation of neuronal and synaptic functionalities constitutes substantial progress toward bioinspired electronics, the development of electronic sensory receptors is crucial for implementing artificial sensory systems. Receptors in sensory systems have vital characteristics that help recognize and process various external environmental stimuli. These characteristics include threshold, adaptation, and sensitivity, collectively contributing to sensory experiences. Volatile memristors offer a feasible means of implementing the characteristics of sensory receptor and neuron, particularly, the threshold. An artificial receptor circuit consisting of a memristive device is presented in Figure 5A. Within the memristor receptor circuit, an externally applied electrical pulse emulates an external stimulus. If the amplitude of this pulse exceeds the threshold voltage of the memristor, the memristor switches to the ON state (LRS), and a current pulse can be observed at the output terminal. This phenomenon corresponds to the perception of stimulus. After this, the memristor reverts back to its original HRS, preparing to react to the subsequent stimulus. In this section, we will discuss the emulation of sensory receptors by implementing a volatile switching memristor. Our focus will be on exploring the methods employed to mimic the functionalities and characteristics of the sensory receptors using memristive devices, which are volatile switching, threshold shift, and adaptation rate.

#### 3.1.1 Volatile switching

Each receptor has a specific threshold, which represents the minimum intensity or concentration of stimulation required to activate and generate a response. Each receptor responsible for each particular sensation operates within its defined threshold, ensuring that only relevant sensory inputs are detected and transmitted to the central nervous system for further processing. Volatile memristors have intrinsic threshold‐switching characteristics, which enable them to transition from a HRS to LRS when subjected to voltages exceeding their threshold, and have been widely developed as sensory receptors.^[^ ^128^, ^129^ ^]^ This property parallels the concept of thresholds in biological receptors, making volatile memristors a suitable technical implementation for mimicking these biological functions.

Moreover, these memristive devices can implement the “relaxation” properties of sensory receptors, which refers to their ability to return to a baseline or inactive state after activation. This property is critical for preventing persistent activation and allowing the receptor to respond to new stimuli.^[^ ^128^ ^]^ Figure 5B explains the threshold characteristics of the volatile memristive device. When an external voltage pulse with an amplitude of *V*_p_ exceeds the threshold value, the device switches from the HRS to LRS after a certain set time (*t*_set_). Upon removing the voltage pulse, the device switches back to the HRS, exhibiting relaxation behaviour for a certain relaxation time (*t*_r_). In the subsequent discussions, we will further explore the details of volatile threshold switching in memristors, factors affecting this switching behaviour, and how these features can be utilized to develop more efficient and biologically realistic artificial sensory receptors.

#### 3.1.2 Threshold shift

Most sensory receptors can adapt to sustained or prolonged stimulation, by adjusting their sensitivity to maintain a dynamic detection range and avoid sensory overload. This crucial adaptation enables receptors to filter out repetitive or irrelevant information while still detecting changes in stimuli, optimizing the interpretation of sensory input. However, in contrast to most other sensory receptors, nociceptors do not adapt to the harmful stimuli they encounter, such as pressure,^[^ ^130^ ^]^ heat,^[^ ^128^ ^]^ or ultraviolet (UV) radiations,^[^ ^131^, ^132^ ^]^ because pain perception is a critical sensory system function that prevents potential or actual tissue damage. These receptors trigger only when the electrical pulse generated by the external stimulus surpasses a specific threshold value, following which they transmit signals to the brain, which are perceived as pain. Upon exposure to harmful stimuli that exceed the threshold, nociceptors reduce their threshold (allodynia) and exhibit a heightened response (hyperalgesia) (Figure 5C). In other words, for injured tissue, the threshold of the nociceptor reduces, and the response intensity increases. This adjustability of the threshold‐switching behaviour by applying excessive electrical stress to the device has been related to receptor sensitization.

Various studies have demonstrated this threshold‐shift behaviour, modelling the sensitization of nociceptors. Yoon et al.^[^ ^128^ ^]^ demonstrated the “sensitization” of their memristive nociceptor (Pt/SiO_x_:Ag/Ag/Pt) by applying high‐amplitude pulses to the devices, emulating damage to the nociceptor system. The threshold voltage shifted to a lower value, and a higher output current was generated, reproducing the allodynic and hyperalgesic characteristics of the nociceptors. The phenomenon of this threshold‐voltage shift could be attributed to residual Ag clusters or conducting filaments, which reduced the effective distance between the top and bottom electrodes and acted as electric‐field concentrators. The effects of reducing the gap and concentrating the electric field of residual Ag clusters could lower the threshold‐switching voltage, mimicking the injury state. Xu et al.^[^ ^130^ ^]^ demonstrated the sensitization behaviour of a memristive device (Ag/copolymer FK‐800/Pt) in response to varying injury pulses and time intervals (Figure 5C). The decrease in the threshold voltage after traumatic injuries corresponded to an increase in the injury pulse amplitude and reduction in the pulse time interval. This implied that not only the injury level but also the elapsed time post injury played an important role in sensitization.

#### 3.1.3 Adaptation rate

The adaptation behaviour of most sensory receptors is characterized by decreasing sensitivity, which enables organisms to filter out unimportant and repetitive information. This behaviour involves sensory processes such as vision, hearing, and touch, and allows the organisms to adapt to changing environmental conditions. Sensory receptors are classified as adaptive or maladaptive based on the rate at which they adapt. Adaptive receptors are activated by harmless stimuli that surpass a specific threshold, and they adjust by reducing their sensitivity, which makes humans discard unnecessary repeated information.^[^ ^133^ ^]^ On the other hand, maladaptive receptors, that is, nociceptors, are activated by stimuli that exceed a high threshold value and they do not adjust or decrease their sensitivity to potentially harmful stimuli.^[^ ^78^ ^]^ Song et al.^[^ ^74^ ^]^ introduced an artificial receptor using a metal–oxide nanorod‐based volatile memristor, designed to emulate both adaptive and maladaptive characteristics. The thickness of the conductive filament was manipulated by varying the amount of Ag metal ions, which govern the balance between Joule heating and electromigration. To compare the adaptation rates of the different devices, a sequence of electrical pulses was applied to memristors with different Ag amounts. Those with less Ag exhibited adaptation behaviour: their output current pulses turned off after a certain number of pulses following an initial current jump (rapid adaptation rate), reflecting the effects of the Joule heating of the thin Ag filament (Figure 5D). Conversely, memristors with a larger amount of Ag formed thicker filaments and maintained their output currents, demonstrating no adaptation to the electrical pulse sequence. These distinct behaviours could be utilized to mimic a selective response (adaptive or maladaptive) to a specific range of external stimulus amplitudes.

### 3.2 Implementation of artificial neuron with memristive devices

Various artificial neuron devices have recently been developed using memristors to generate spike signals without the assistance of complex circuits. Although non‐volatile memristors have also been developed as artificial neurons,^[^ ^90^, ^135^, ^136^ ^]^ volatile memristor neurons are the most widely developed devices for artificial neurons. Compared to volatile memristors, there are several limitations when applying non‐volatile memristors to artificial neurons. First, the rapid computation may be limited since non‐volatile memristors generally have a slower switching speed than volatile memristors. In addition, due to its non‐volatile nature, although less reset or loss of information occurs in unexpected situations, additional mechanisms or strategies may be required to erase or update old information. These shortcomings can be important considerations, especially in research and applications related to the implementation of artificial neurons. Therefore, various studies and developments are underway to solve these problems. However, as few studies are related to the application to artificial sensory systems, this review focuses on artificial neurons based on volatile memristors. Extensive research has been conducted using the emerging memristor technology.^[^ ^46^, ^50^, ^137^, ^138^ ^]^ Artificial neurons must possess the fundamental characteristics that enable them to accumulate signals and generate spikes once the stimulus intensity surpasses a threshold. Therefore, the memristor needs specific characteristics such as threshold and relaxation behaviour, energy‐efficient operation, and rapid response. The volatile memristor satisfies these characteristics, and numerous studies have successfully demonstrated the feasibility of artificial neurons based on volatile memristors, using various mechanisms.

#### 3.2.1 Leaky integrate‐and‐fire

The threshold function of neurons determines whether sufficient signals have been collected from the synapses. When a biological neuron receives a certain level of signal from a previous neuron through dendrites, the neuron is activated and transmits the signal to the next neuron through axons. Various neuron models such as the Hodgkin–Huxley^[^ ^139^ ^]^ and Izhikevich^[^ ^140^ ^]^ models, have been proposed to simulate the behaviours of these biological neurons. Among them, the integrate‐and‐fire (IF) and leaky integrate‐and‐fire (LIF) neuron models have been studied as strong candidates. Although the IF and LIF models have low biological realism, they are more widely used in neuromorphic computing systems owing to their computational efficiency. Therefore, we will mainly introduce the IF and LIF models and their memristor‐based implementations.

**FIGURE 6:**
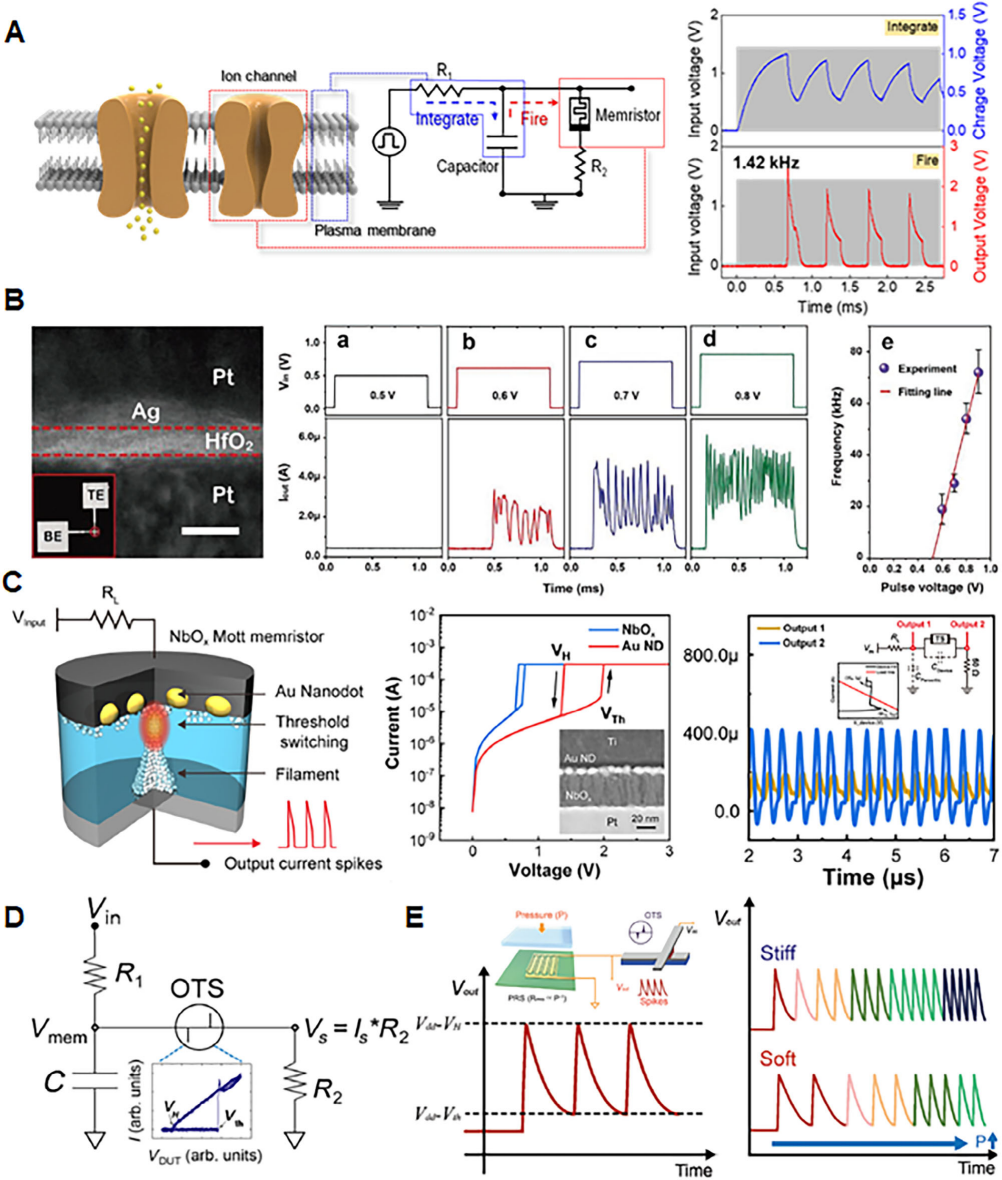
(A) Schematic illustration of the leaky integrate‐and‐fire (LIF) spiking circuit (left). The voltage across the capacitance (blue curve) and output firing (red curve) under a train of input voltage pulses (grey curve) (right). Reproduced with permission.^[^ ^180^ ^]^ Copyright 2023, American Chemical Society. (B) Cross‐sectional transmission electron microscopic image of Ag‐nanodot (ND)–introduced device (left). Oscillation current responses to different input pulses (right). Reproduced under the terms of the Creative Commons CC BY license.^[^ ^154^ ^]^ Copyright 2019, The Authors, published by WILEY‐VCH Verlag GmbH & Co. KGaA, Weinheim. (C) Schematic of the spike generator system using Ti/Au ND/NbO*_x_*/Pt device (left). Current–voltage (*I−V*) curves of the Ti/NbO*_x_*/Pt device and Au ND device (centre). Self‐oscillation of NbO*_x_* threshold‐switching devices (inset: schematics of oscillation circuit) (right). Reproduced with permission.^[^ ^159^ ^]^ Copyright 2023, American Chemical Society. (D) Artificial ovonic threshold switching (OTS) neuron circuit composed of two resistors (R1 and R2), one capacitor, and one OTS device. (inset) *I–V* curves of the OTS device. Reproduced with permission.^[^ ^177^ ^]^ Copyright 2022, Wiley‐VCH GmbH. (E) Spike generated by the OTS neuron integrated with piezosensitive sensor (left). Spike evolution patterns according to different levels of elastic stiffness of the contacting material (right). Reproduced with permission.^[^ ^179^ ^]^ Copyright 2022, Wiley‐VCH GmbH.

Figure 6A presents a schematic diagram of a circuit constructed using threshold‐switching devices and manual components, to simulate the behaviour of biological neurons. The resistor and capacitor connected in front of the threshold‐switching device integrate the signals received from the dendrites. The threshold‐switching device mimics the firing of biological neurons, changing from the HRS to LRS when a specific critical voltage is reached. The threshold‐switching device is initially at the HRS; therefore, even if a voltage is applied, the leakage current through the device is small and charges accumulate in the capacitor, which increases the membrane voltage. When the membrane voltage rises to the threshold voltage of the threshold‐switching device, the device changes to the LRS, and the charges accumulated in the capacitor exit through the device, generating an output spike signal. As the charge accumulated in the capacitor escapes, the membrane voltage decreases again. As a result, the threshold‐switching device returns to the HRS, repeating the operation of accumulating charges in the capacitor.^[^ ^50^ ^]^ To emulate the behaviour of biological neurons, studies on threshold‐switching devices using Ag‐based memristors, metal–insulator transition materials, and chalcogenide materials have been reported.

#### 3.2.2 Ag‐based memristors for artificial neurons

The phenomenon of resistive switching results from the diffusion and migration of active metal ions like Ag and Cu in dielectrics. The duration for which these mobile ions maintain a conductive pathway between the top and bottom electrodes determines the categorization of the device, either as resistive switching^[^ ^141^, ^142^, ^143^ ^]^ or threshold switching. For example, the retention time in Ag filament‐based resistive switching memory device devices is determined by the diffusion of Ag once the filament is formed.^[^ ^142^ ^]^ Zhang et al. demonstrated that as the lattice size gets smaller, the energy barrier for Ag diffusion increases, leading to a balance between device switching speed and its retention duration.^[^ ^142^ ^]^ In addition, according to Ag doping concentration, Im et al. have proposed the mechanism of volatile and non‐volatile behaviour of Ag conductive filament in a switching matrix.^[^ ^143^ ^]^ They offered an efficient strategy to integrate the Ag‐based bidirectional threshold and bipolar resistive switching, which can be coupled into the 1S‐1R integrated component. In contrast, because of the diffusive nature of Ag conductive filament, diverse Ag‐based diffusive memristors act as artificial neuron devices with volatile threshold switching properties and excellent ion‐migration dynamics across insulating layers (SiO_2_, HfO_2_, or Ta_2_O_5_). With the application of an appropriate electric field, Ag diffuses from the upper electrode through the switching channel, forming a conductive path between the upper and lower electrodes. When the electric field is removed, the Ag filament voluntarily turns into a sphere to minimize the interfacial energy. This behaviour ruptures the conductive filament, facilitating relaxation of the device and enabling volatile behaviour, making it the most suitable candidate for mimicking thresholds in biological neurons.

Based on an Ag/SiO_2_/Au threshold‐switching memristive device, Zhang et al.^[^ ^92^ ^]^ reported an IF neuron device with four critical neuron features: the all‐or‐nothing spiking of an action potential, threshold‐driven spiking, refractory period, and strength‐modulated frequency response. This IF neuron device acted as a post‐neuron to integrate the signals received as inputs via a capacitor and generate spiking pulses. When the applied voltage exceeded a threshold value, the device switched to the LRS by forming Ag filaments, and when the applied voltage was lower than a hold voltage, the device spontaneously reverted to the HRS by regrouping Ag atoms to prepare the next fire action. Finally, they demonstrated that this Ag‐based neuron device was applicable to digit recognition, verifying its feasibility for application in SNNs.

Wang et al. reported LIF neurons doped with Ag nanoclusters in SiO*_x_* N*_y_* and integrated them with crossbar‐array–type synapses to create a neural network.^[^ ^144^ ^]^ The internal Ag dynamics of the memristor generated the output voltage, by interacting with the RC time constant of the circuit. With a smaller capacitance, the RC time became shorter, and the internal Ag dynamics of the memristor dominated the delay and thus, the IF behaviour. The neural network consisting of eight neurons and 8 × 8 synapses successfully classified four‐character patterns (“U,” “M,” “A,” and “S”) with artificially added noise, through different time‐to‐time current responses. This report showed that oxide‐based neuron device arrays could infer and recognize, achieving energy‐efficient learning, and that more complex learning could be achieved through system expansion.

The internal Ag dynamics of memristors result from complex multiphysical effects, including Ag mass transfer (e.g., Ag diffusion and redox reactions) and the formation of electrical conduction paths from electrodes, which can improve the properties of neuron devices.^[^ ^145^, ^146^, ^147^, ^148^, ^149^ ^]^ Owing to the stochastic dynamics of Ag filament growth, which inherently reduce the significant cycle‐to‐cycle or device‐to‐device variations, Ag‐based threshold‐switching memristors should be improved for practical application in neuromorphic systems. In addition, it is difficult to achieve high current density, using Ag‐based threshold‐switching devices, because voltage or current must be suppressed (compliance current) so that thick Ag filaments with non‐volatile properties are not formed by overvoltage and overcurrent. Recently, some strategies aimed to enhance Ag controllability and achieve high current density by confining Ag migration were proposed, including dielectrics engineering through grain‐boundary orientation,^[^ ^150^ ^]^ filament confinement within a small area via nanowires,^[^ ^151^ ^]^ embedding Ag nanowires in a polymer,^[^ ^152^ ^]^ or using highly ordered Ag nanodots.^[^ ^153^ ^]^ The following section introduces studies implementing Ag‐based threshold‐switching devices as artificial neuron devices with improved characteristics by applying Ag dynamics‐regulated elements.

Hua et al.^[^ ^154^ ^]^ achieved an adequately large on‐state current on Ag nanodots/HfO_2_‐based threshold‐switching devices through the introduction of highly ordered Ag nanodots as active electrodes (Figure 6B). Compared to using the conventional Ag film, using Ag nanodots as active electrodes could prevent excessive Ag migration into the switching matrix during operation, resulting in stable conductive filament growth. The Ag nanodot‐based threshold switch exhibited bidirectional electroforming‐free operation with ultralow leakage current (<1 pA) and large on‐state current (500 μA). Moreover, the rapid thermal processing (RTP)‐treated Ag nanodot/HfO_2_ device showed superior threshold‐switching performances, as this thermal treatment enhances Ag diffusion into the matrix and accumulation on the bottom electrode/HfO_2_ interface, resulting in multiple weak Ag filaments. As shown in Figure 6B, the self‐oscillation characteristics of the device were measured by increasing the input pulses. In addition, by using a nanocontact structure strategy, Wang et al.^[^ ^152^ ^]^ demonstrated an improved threshold‐switching device with high non‐linearity (10^10^) and high on‐state current (500 μA). The device in their study (Ag/Ag nanowires/silk or PDMS/Au) was based on a nanocomposite thin film consisting of an insulating matrix and Ag nanowires, enabling flexibility and forming a nanoscale conductive filament via the nanocontact effect. This nanoconfinement effect of Ag nanowires, which formed a filament retaining large interfacial energy, was attributed to the reliable and non‐linear threshold‐switching behaviour. The above studies suggested that the spatial confinement of Ag migration was an important factor for improving the threshold‐switching characteristics.

#### 3.2.3 Metal–insulator transition‐based memristor for artificial neurons

Mott materials are materials whose resistance states change by the metal–insulator transition (MIT). According to the density functional calculation of the band structure, Mott materials must have metallic properties. However, owing to the strong Coulomb repulsion of electrons, they show insulating properties.^[^ ^155^, ^156^ ^]^ AM_4_Q_8_ (A = Ga, Ge; M = V, Nb, Ta, Mo; Q = S, Se) structures exhibit Mott transition properties because of the presence of Mott–Hubbard gaps in transition metals.^[^ ^157^ ^]^ Oxide‐based materials such as VO_2_ and NbO_2_ also show Mott transition properties, mainly because of the negative‐differential‐resistance effects.^[^ ^158^ ^]^ When an external voltage bias is applied, current flows through a specific pathway in the device, leading to Joule heating. As the Mott transition temperature exceeds a specific threshold induced by a sufficiently large current, it transitions from an insulator to a metal phase. However, the metallic phase dissipates upon removing the external bias and eliminating heat, resulting in the memristor returning to its insulating state.

Research on using Mott transition characteristics in artificial neuron devices is actively being conducted. As MIT‐based neuron devices switch between the insulating and metallic phases because of the atomic rearrangement, they have higher switching speeds owing to the short‐range atomic rearrangement (≈ns),^[^ ^98^, ^159^ ^]^ making them suitable for high‐frequency spike generators. Owing to their high switching speeds, which follow the increment in charging speed, they show linear increases in spikes with the increase in the input current amplitude. Compared to neurons based on other mechanisms, MIT‐based neurons have lower *R*_off_, inducing fast discharging speed with leaky characteristics.^[^ ^97^ ^]^ However, as they require significant operating currents (≈mA) to reach the phase‐transition temperature (≈1080 K), the energy consumption of MIT‐based neurons is greater than that of Ag‐based neurons.

Based on the highly reliable spike‐generation property of the NbO_2_ memristor, Zhang et al.^[^ ^18^ ^]^ demonstrated an artificial spiking afferent nerve, which generated a spiking frequency proportional to the intensity before encountering ordinary stimuli. The NbO*_x_*‐based neuron used in this study could transform analog pressure signals into reliable oscillation frequencies, which exhibited quasi‐linear relationships with the input voltage. The increasing frequency decreased when the generated voltage strength became extremely high, closely resembling the operation of a biological neuron. This frequency reduction could be explained by considering that the relaxation time of the NbO*_x_* memristor would be extended under high input intensities. Eventually, the oscillation period would be dominated primarily by the relaxation time.

A high amplitude output spike is necessary for accurate signal detection and noise‐tolerant signal transmission for utilizing MIT‐based neurons for sensory perception application. Though the spike amplitude can be modulated using external electrical components, it could produce a complexity issue at the circuit level. Park et al. demonstrated a material‐based approach to achieve high‐amplitude spikes by incorporating Au nanodots into the NbO_2_ device (Ti/Au nanodots (ND)/NbO*_x_*/Pt).^[^ ^159^ ^]^ The presence of Au NDs allowed for the modulation of oxygen content at the interface between the electrode and oxide in the devices, reducing the filament size and resulting in a higher ON current (six times higher) compared to Au ND‐free devices without any additional external circuit element (Figure 6C). Moreover, as the threshold‐switching mechanism in MIT‐based devices highly relied on the temperature from Joule heating, the heat confinement effect in the switching matrix had a significant influence on the device properties. In this work, a higher Joule heat was generated by inducing a higher ON current through a thinner filament, making the crystallization of NbO_2_ longer along the vertical direction and resulting in a more extended threshold‐switching region.

Handling multimode physical signals with high uniformity in neuromorphic perception systems is incredibly desirable in neuron devices. Yuan et al. reported calibratable neuron devices of epitaxial VO_2_ memristors grown via pulsed laser deposition.^[^ ^160^ ^]^ The improved cycle‐to‐cycle uniformity of the neuron device was achieved using the high crystalline quality of epitaxial VO_2_. Moreover, the oscillator period could be varied according to the degree of stimulation by connecting the VO_2_‐based device and detecting pressure, light, and temperature. They integrated the curved sensor with a finger to confirm that the frequency of the VO_2_‐based oscillator varied depending on the degree of bending. Thereby, they demonstrated the possibility of its use in wearable equipment.

Despite successful research results using Mott materials, the development of MIT‐based neuron devices has been limited by the operation‐temperature constraints imposed by the phase‐transition temperature of Mott materials. An alternative and promising solution lies in applying ovonic threshold switch (OTS) devices, which rely on an electrical‐switching mechanism described by the trap‐mediated excitation of carriers followed by an avalanche effect. Therefore, exploiting the advantages of OTS devices can help overcome the limitations of Mott‐material–based devices and further advance the development of efficient and practical artificial neuron devices. In the following section, we will introduce the operating mechanism of the OTS device and its application as an artificial neuron.

#### 3.2.4 Ovonic threshold switching‐based memristor for artificial neurons

OTS devices, which have a metal–amorphous‐chalcogenide–metal structure, exhibit high operation speed,^[^ ^161^ ^]^ and excellent endurance characteristics,^[^ ^162^, ^163^, ^164^, ^165^ ^]^ satisfying the crucial requirements for neuron devices. By optimizing the elemental ratios and concentrations of components, the thermal stability and endurance characteristics of the OTS devices can be improved.^[^ ^166^, ^167^ ^]^ For example, Garbin et al. showed stable endurance characteristics for more than 10^11^ cycles by optimizing the elemental ratio of As/Te in a Si‐Ge‐As‐Te OTS material.^[^ ^162^ ^]^ Moreover, OTS devices show excellent compatibility with the conventional CMOS processes, as evidenced by their successful application in commercial devices. Although OTS devices offer many advantages in terms of the performance, their switching mechanism still needs to be determined. A variety of theoretical models have been proposed to explain the OTS phenomenon, such as thermally induced instability,^[^ ^168^ ^]^ Shockley–Read–Hall recombination with impact ionization,^[^ ^169^, ^170^ ^]^ polaron destabilization,^[^ ^171^ ^]^ nucleation theory,^[^ ^172^ ^]^ and thermally assisted hopping model.^[^ ^173^ ^]^ However, none of the proposed mechanisms could fully account for the observed characteristics, and no unified model could represent the physical switching of OTS. Nevertheless, the consensus among most models was that the OTS operated primarily via an electronic phenomenon, supplemented by a secondary thermal effect not involving the atomic arrangement.

Based on the threshold‐switching characteristic, the oscillating behaviour of artificial neurons can be implemented using an OTS device exhibiting reversible electrical switching.^[^ ^173^, ^174^, ^175^, ^176^ ^]^ Figure 6D presents an artificial OTS neuron circuit composed of two resistors (R1 and R2), one capacitor (C), and one OTS device. The following section will introduce the implementation of the OTS device as an artificial neuron device presenting IF behaviour. Lee et al. introduced an artificial neuron based on OTS with promising features for mimicking the complex behaviour of biological neurons, such as spike‐frequency adaptation, chaotic activity, and LIF behaviour.^[^ ^177^ ^]^ They used a cross‐point–structured OTS device with Ge_60_Se_40_ as an active switching material (Mo/Ge_60_Se_40_/Mo) and a few passive electrical components. They successfully performed a spoken‐digit recognition test using their OTS‐based neuron, resulting in high recognition accuracy. They also demonstrated a three‐terminal OTS (3T‐OTS) device based on GeSe_2_, which featured an electrically controllable threshold voltage.^[^ ^178^ ^]^ As the electric field was determined by the thickness, the threshold voltage also depended on the thickness of the switching films. Especially, the atomic ratios of the components determined the threshold voltage of the OTS device, as a reduction in the number of components decreased the number of traps. In their study, the threshold voltage was modulated by the gate voltage, and this threshold‐voltage control enabled gate‐voltage–induced modulation of the spike frequency of the artificial neurons. Moreover, when a photodiode was connected to the gate electrode, this 3T‐OTS device generated spikes at a frequency that depended on the external light stimuli, similar to a biological retinal ganglion cell. As shown in Figure 6E, Lee et al. also developed a stiffness‐encoded artificial spiking tactile neuron based on an OTS device, connecting a piezoresistive sensor as an artificial mechanoreceptor with high endurance characteristics (>10^5^).^[^ ^179^ ^]^ With this configuration, the pressure level was encoded into spikes at a frequency proportional to the intensity of the external pressure.

Hwang et al. investigated the effects of OTS device characteristics, in comparison with that of an Ag‐based atomic‐switching device and a NbO_2_‐based MIT device.^[^ ^97^ ^]^ Table 1 compares the device features associated with neuron behaviour, using three types of threshold‐switching devices. The mechanism of each device type determines the switching speed. In other words, unlike Ag‐based devices with low switching speeds (≈μs) based on a mechanism involving slow atomic movement, MIT devices with short‐range atomic‐rearrangement–based switching mechanism and OTS devices based on electronic phenomena without atomic rearrangement show faster switching speeds (≈ns). In addition, Ag‐based neurons show slower delay times (off‐to‐on state) and relay times (on‐to‐off state) than the MIT‐ and OTS‐based neurons, because of their ionic motion mechanism. As the discharging speed and current in the integration process determine the energy‐consumption efficiency, MIT devices with discharging speeds greater than that of other neurons, because of the low off‐resistance (at 0.5 threshold voltage, the off‐current values of MIT, OTS, and Ag‐based neurons are found to be approximately 3 μA, 5 nA, and 1 pA, respectively), exhibit higher power consumptions in the fire‐and‐reset process. Relevant research on the use of each of the three neuron devices is summarized in Table 2.

**TABLE 1:** Comparison of device characteristics of three different types of neuron devices, in terms of neuron behaviour.^[^ ^97^ ^]^

Neuron device	Ag‐based ionic switching device	Ovonic threshold switching (OTS) device	Metal–insulator transition (MIT) device
Mechanism	Formation & dissolution of unstable metal filament made injected active electrode	Electronic phenomenon with secondary thermal effect	Atomic rearrangement between insulating and metallic phases by field‐induced Peierls phase
Switching speed	Low (≈μs) (because of slow ionic movement)	High (≈ns)	High (≈ns) (because of short‐range atomic rearrangement)
# of spikes as amplitude of *I*_input_ increase	Spike saturation (because of low switching speed, which cannot follow the increase in charging speed)	Linear increase (because of high switching speed, which can follow the increase in charging speed)	
Activation function	Sigmoid function	Rectified linear unit (ReLU) function	
Threshold voltage	Related to material parameters (diffusivity of mobile ions)	Dependent on atomic ratio of components	Dependent on thickness of switching films (because electric field is determined by thickness)
Discharging speed	Slow (because of extremely large *R*_off_) → Nonleaky	Slow (because of large *R*_off_) → leaky	Fast (because of small R_off_) → leaky
Threshold current for firing	Low (because of slow discharging speed)	Medium	High (because of fast discharging speed)
Energy consumption	Low (because of low discharging current)	Medium	High (because of high discharging current)
Delay time (off‐to‐on state)	Slow (e.g., Ag/HfO_2_ ≈ 10 μs)	Fast (e.g., B‐Te ≈ 7 ns)	Fast (e.g., NbO_2_ < 30 ns)
Recovery time (on‐to‐off state)	Slow (e.g., Ag/HfO_2_ ≈ 10 μs)	Fast (e.g., B‐Te < 10 ns)	Fast (e.g., NbO_2_ < 10 ns)

**TABLE 2:** Summary of artificial neurons demonstrated with three types of memristors.

Mechanism	Structure	Operating voltage	Energy per spike	Max. frequency	Ref
Ag‐based	Ag/SiO_2_/Au	1.2 V	—	25 Hz	^[^ ^92^ ^]^
	Pt/SiO*_x_* N*_y_* Ag/Pt	0.5 V	—	—	^[^ ^144^ ^]^
	Pt/Ag nanodots/HfO_2_ /Pt	0.6 V	—	72 kHz	^[^ ^154^ ^]^
	Ag/SiO_2_ (with protein nanowire)/Pt	0.1 V	0.1 pJ	—	^[^ ^181^ ^]^
	Ag/HfO_2_/Pt	0.3 V	0.27 pJ	—	^[^ ^97^ ^]^
	Ag/Mxene/SiO_2_/Pt	0.18 V	—	—	^[^ ^182^ ^]^
MIT	Ti/Au nanodots/NbO*_x_*/Pt	0.8 V	—	3.14 MHz	^[^ ^159^ ^]^
	Au/Ti/VO_2_/c‐Al_2_O_3_	1.35 V	2.9 nJ	1.3 MHz	^[^ ^160^ ^]^
	TiN/NbO*_x_*/poly‐Si	2.05 V	38 pJ	10 kHz	^[^ ^18^ ^]^
	Pt/NbO_2_/Pt	1.9 V	—	33 MHz	^[^ ^183^ ^]^
	W/NbO_2_/W	1.1 V	50 pJ	0.45 Hz	^[^ ^97^ ^]^
	Pb/NbO_x_/Pt	2.2 V	0.9 nJ	2.67 MHz	^[^ ^36^ ^]^
	Pt/TiN/NbO_2_/TiN/W	2.1 V	2 pJ	9 MHz	^[^ ^184^ ^]^
OTS	Mo/Ge_60_Se_40_/Mo	5.4 V	1.2 nJ	65 MHz	^[^ ^177^ ^]^
	Au / TiN /Ag*_x_* (GeSe_2_)_1−_ *_x_*/Pt	1.8 V	—	0.26 MHz	^[^ ^178^ ^]^
	Pt/Sn_13_Ge_37_Se_50_/TiN	2.8 V	3.54 nJ	1.01 MHz	^[^ ^179^ ^]^
	Al/TiN/GeS/W	‐3.2 V	—	7 MHz	^[^ ^185^ ^]^
	W/B‐Te/W	0.7 V	—	0.45 Hz	^[^ ^97^ ^]^

### 3.3 Implementation of artificial synapse with memristive devices

To emulate the functionality of a synapse that memorizes responses to external stimuli, a memristor must meet certain characteristics. It must exhibit non‐volatile properties so that it can save its state even after the applied voltage is removed. In addition, when the resistance state of the device changes according to the external voltage, the changed behaviour can act as an important parameter when implementing higher intelligence using synaptic plasticity. Thus, memristors are important for effectively mimicking the complex properties observed in biological synapses. In this section, we discuss the essential requirements that must be met to successfully mimic synaptic properties using memristors.

#### 3.3.1 Non‐volatile switching

The synaptic connection is influenced by the strength of the input signal received from the axon of the pre‐neuron. This strength, commonly known as synaptic weight, undergoes potentiation or depression in response to the input stimuli, a process referred to as synaptic plasticity. The resulting scaled output signal is then transmitted to the dendrite of the post‐neuron. Non‐volatile switching memristors have been proposed as a potential solution to serve as synaptic devices. Memristors exhibit non‐volatile properties, owing to various effects such as redox reactions (electrochemical metallization (ECM) and valance change mechanism (VCM),^[^ ^186^, ^187^, ^188^, ^189^, ^190^, ^191^ ^]^ thermal effects (thermochemical memory (TCM),^[^ ^192^, ^193^ ^]^ and phase‐change memory (PCM)^[^ ^194^, ^195^, ^196^, ^197^ ^]^) and ferroelectricity (ferroelectric tunnel junction (FTJ) and ferroelectric field‐effect transistor (FeFET)^[^ ^198^, ^199^, ^200^, ^201^ ^]^). ECM and VCM based on redox reactions change the resistance by forming conductive filaments based on metal and oxygen deficiency, respectively. TCM utilizes the thermal effects observed when a thermochemical redox process dominates the electrochemical process, exhibiting a primarily unipolar transition. PCM relies on thermal effects that induce phase changes within the phase‐change material by Joule heating upon the application of electrical pulses. FTJ and FeFET, which are based on ferroelectric materials, exhibit resistance changes because of the influence of polarization when the applied voltage surpasses the coercive field (*E*_c_).

Redox‐based memristors such as ECM and VCM offer distinct advantages over other types of memristors, and allows effective manipulation of redox reactions with controllable redox rates, activation energies (*E*_a_), and ion mobilities by using various materials and structures. This capability has led to active research on enhancing the essential characteristics required for the implementation of artificial synapses, involving synaptic weight control, switching speed, uniformity, retention, and endurance.^[^ ^202^, ^203^, ^204^, ^205^ ^]^ Especially, because of their low operating voltage^[^ ^206^, ^207^ ^]^ and rapid resistance transition,^[^ ^208^, ^209^, ^210^ ^]^ redox‐based memristors can achieve lower power consumptions, compared to that of the other mechanisms. Moreover, they offer improved scalability by facilitating the creation of nanoscale conductive pathways in conductive filament, which is crucial for developing high‐density, high‐capacity synapse arrays and large‐scale neuromorphic computing systems.^[^ ^211^ ^]^ Therefore, we will focus on redox‐based memristors that are highly suited for implementing artificial synaptic properties.

Similar to biological synapses, non‐volatile memristors function as memory elements, storing the strength of synaptic connections even in the absence of power. However, to fully replicate the behaviour of synapses, the non‐volatile memristors must satisfy specific criteria such as variation, retention, endurance, on/off ratio, multilevel state, and linearity of conductance modulation. These characteristics are crucial for accurately reproducing synaptic functionalities in artificial systems. The multilevel state and linearity of conductance modulation will be discussed in the following subsection.

**FIGURE 7:**
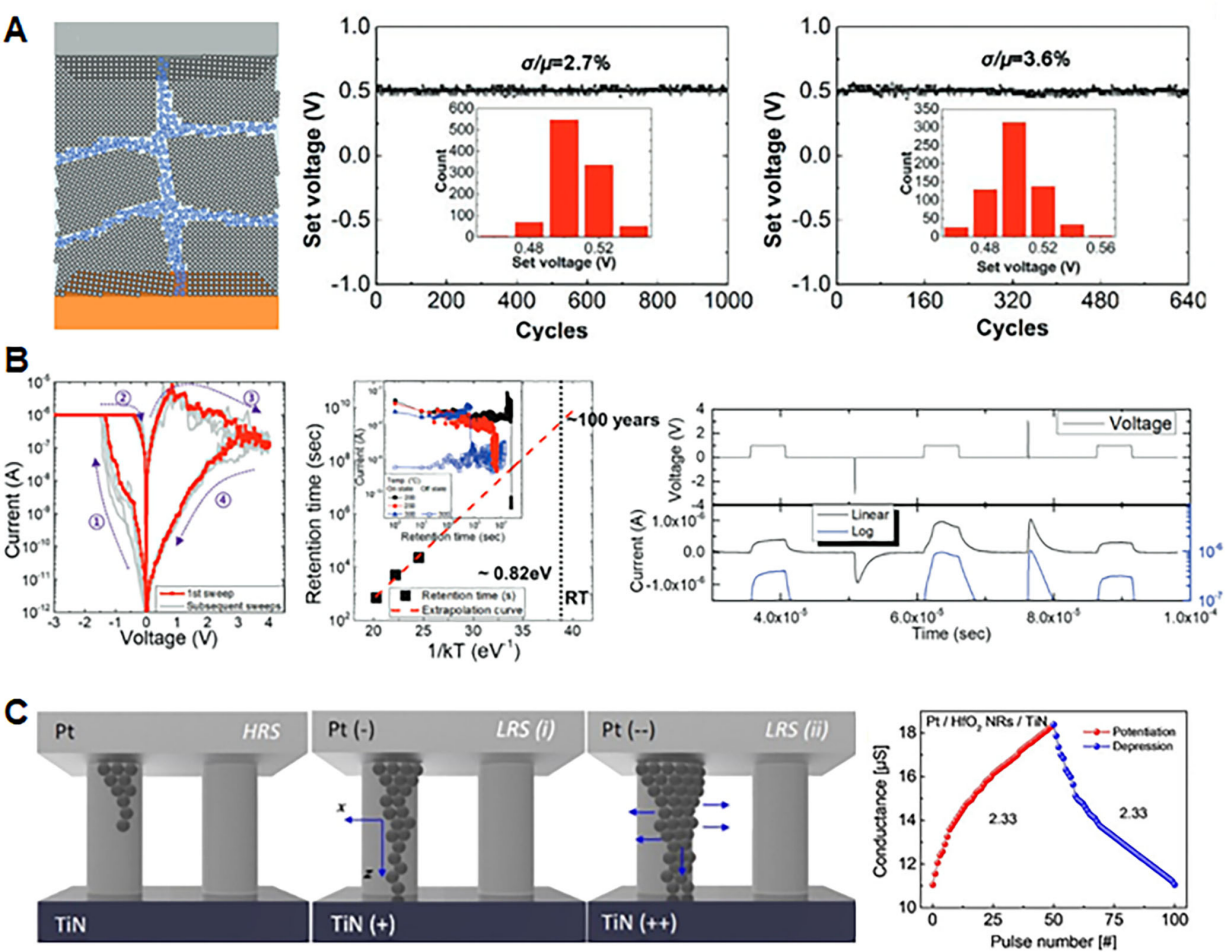
(A) Schematic illustration of the phase‐separated oxide (PSO) memristor (left). The set voltage variation of the PSO‐memristive device after 1000 current–voltage (*I–V*) sweeps (middle) and all 32 devices (right). The inset histogram represents the set voltage distribution. Reproduced with permission.^[^ ^228^ ^]^ Copyright 2020, Wiley‐VCH GmbH. (B) *I–V* curves of Pt/Ta_2_O_5_/Ru device showing resistive switching behavior (left). The time‐failure graph of the device and an inset graph showing the retention time of the off‐state and on‐state at different temperatures (middle). Schematic graph of the input voltage pulses for read–set–read–reset–read process and the output current of the device corresponding to the input voltage pulses shown in both the linear and log scales (right). Reproduced with permission.^[^ ^229^ ^]^ Copyright 2020, WILEY‐VCH Verlag GmbH & Co. KGaA, Weinheim. (C) Schematic of the conductive filament growth mechanism in nanorod‐based memristors (left; HRS: high‐resistance state; LRS: low‐resistance state). Uniform repeatability of potentiation and depression characteristics in the case of the nanorod‐based memristors. Reproduced with permission.^[^ ^254^ ^]^ Copyright 2022, American Chemical Society.

First, durability (endurance and uniformity) is essential for a stable switching behaviour. In neuromorphic computing, memristors are used to emulate synaptic connections between neurons. To perform accurate and precise computations, the behaviour of each memristor must be consistent and uniform. Notable variations in properties such as resistance levels or switching characteristics among memristors can introduce inconsistencies and compromise the accuracy of the computations performed by the neural network. However, in conventional ECM or VCM‐based memristors, the random formation and rupture of conductive filaments result in significant temporal and spatial variations.^[^ ^212^, ^213^, ^214^, ^215^, ^216^ ^]^ To address these limitations, reducing the stochasticity of filament formation and rupture is critical. Numerous researches have focused on mitigating this randomness. One approach established a defined pathway that promoted filament formation, thereby controlling the area where filaments were generated.^[^ ^217^, ^218^, ^219^ ^]^ Another method involved managing the quantity of filament‐forming sources to control the variability caused by excessive generation.^[^ ^220^, ^221^, ^222^ ^]^ Additionally, doping specific elements into oxide thin films was explored as a way to reduce ion oxidation and reduction.^[^ ^223^, ^224^, ^225^, ^226^, ^227^ ^]^ These studies presented various techniques and strategies for adjusting and optimizing these factors. For example, Wan et al. suggested the use of memristors composed of engineered micro‐ and nanostructures with modified compositions and vertical hetero‐interfaces.^[^ ^228^ ^]^ As shown in Figure 7A, the microstructure of the film consisted of amorphous TiO_2_ channels as the switching medium and neighbouring crystalline CoO grains as oxygen reservoirs. This configuration allowed the confinement of the conductive filaments within localized areas, enabling better control over oxygen‐vacancy transport, and reducing the over‐injection of vacancies. Hence, the memristor produced significant improvement by eliminating the need for an electroforming process, a soft breakdown process characterized by high voltage requirements and the associated variability. Given the high voltage applied, it can induce structural deformation of the device (such as the formation of oxygen bubbles) and potentially cause a permanent breakdown. This process necessitates additional peripheral circuits for operation and demands high power consumption. Furthermore, from the perspective of device variation, random filament formation can be a source of device‐to‐device variation. Therefore, eliminating the electroforming process makes it possible to address the device variation issues, enabling the fabrication of high‐reliability devices, and achieving lower energy consumption.

Secondly, efficiently replicating the processing capabilities of the human brain using memristors necessitates low energy consumption despite the need to handle significant amounts of data. To achieve this, the memristor must exhibit high operating speeds at low voltages and currents. However, reducing the operating voltage and current levels can suppress the device's ability to maintain stable conductive states, leading to reliability issues. If a conductive filament is formed with relatively low energy, it will be more prone to be weak, which increases the likelihood of spontaneous or undesired rupture, particularly, without biasing or when additional voltage is applied. Researchers are actively exploring solutions to overcome this inherent trade‐off.^[^ ^229^ ^]^ In the conventional VCM devices, the large activation energy required for oxide diffusion makes achieving low switching voltage and current, simultaneously, difficult. On the other hand, ECM devices utilizing cations, having lower activation energies for oxide diffusion, suffer from poor retention because of the low activation energy for diffusion. To overcome these limitations, there is a growing demand for novel mobile species that can combine the advantages of both VCM and ECM. Identifying a new mobile species with characteristics such as suitable activation energy and mobility makes it possible to achieve a trade‐off between switching voltage/current and holding time. This intermediate species, positioned between oxygen vacancies and metal ions, is a promising solution to overcome these limitations. For example, the Ru‐ion–mediated ECM‐like device exhibits a median activation energy of approximately 0.82 eV, which limits the movement of Ru and causes noncontinuous CF (nanoclusters) at low compliance currents. In contrast to the continuous metal CF that exhibits high‐current operation, the Ru‐based nanocluster memristor operates based on a tunnelling conduction mechanism with a low‐current operation. Therefore, it enables the realization of both low switching voltage/current and good retention in a single device, as shown in Figure 7B.

Finally, the analog switching behaviour of memristors, which enables them to store and adjust continuous values of resistance or conductance, is crucial for simulating artificial synapses. This feature is essential for emulating artificial synapses with variable ranges of synaptic weights. Unlike the abrupt switching with limited states (on and off), the analog switching behaviour offers a more accurate representation of synaptic weights. In other words, they allow fine‐grained modulation, facilitating precise adjustments and updates to synaptic strength. This analog behaviour enhances the fidelity and flexibility of the artificial neural networks, enabling efficient information processing and learning.^[^ ^230^ ^]^

#### 3.3.2 Conductance modulation and linearity

The linear relationship between the synaptic weight change (∆*w*), programming pulse, and multilevel states is important for mimicking the biological synapses. The change in conductance is utilized to estimate the intensities of the previous input signals, making the linear‐conductance change particularly significant as it enhances the accuracy of inferring the intensities of these signals. Linearity refers to the proportionality between an input signal applied to a synapse and the resulting change in conductance. In other words, a linear relationship means that a small change in the input signal will lead to a proportional change in the conductance value. This linearity allows for fine‐grained control over the synaptic weights and facilitates precise adjustments during learning and information processing. Furthermore, linearity enables the straightforward mapping of mathematical operations onto neuromorphic hardware. Many algorithms and learning rules in machine learning and neural networks are designed based on linearity assumptions. By preserving linearity in conductance modulation, neuromorphic systems can leverage the existing algorithms and take advantage of the rich body of knowledge collated in the field of traditional computing. Conductance modulation that exhibits a linear relationship is essential to establish memristor‐based neural networks.^[^ ^64^, ^231^, ^232^, ^233^, ^234^, ^235^ ^]^ Numerous techniques have been developed to achieve the linearity of conductance modulation. Some methods that control the generation path of conductive filaments by utilizing interfaces,^[^ ^236^, ^237^ ^]^ grain boundaries,^[^ ^238^, ^239^ ^]^ the growth of conductive filaments by doping metals in the electrolyte layer,^[^ ^240^, ^241^, ^242^, ^243^, ^244^ ^]^ and use nanostructures.^[^ ^245^, ^246^, ^247^, ^248^, ^249^, ^250^, ^251^ ^]^

Ryu et al. demonstrated a method for achieving high linearity utilizing a bilayer structure (W/Al_2_O_3_(3 nm)/HfO_2_(7 nm)/TiN) and optimized measurement schematic.^[^ ^252^ ^]^ The presence of Al_2_O_3_ in the device structure played a significant role in influencing the electronic energy barrier during SET and RESET. By adopting a bilayer structure, the CF in the memristor was ruptured near the interface (acting as the energy barrier) between the Al_2_O_3_ and HfO_2_ layers during the reset process, while the residual CF remained in the HfO_2_ layer. This bilayer structure limited the CF rupture and formation region, contributing to the overall functionality of the device and improving the switching behaviour. The intrinsic symmetric operating voltage and optimized electrical pulse schemes for both potentiation and depression are crucial factors for achieving reliable synaptic performance. While conductive‐filament–based memristive devices with self‐compliance and symmetric operating voltages are capable of achieving reliable synaptic performance, it is important to note that the weight modification can be overestimated if the fluctuations in weight variation are ignored.

Kang et al. suggested an approach that regulated the probability of cation reduction during the switching process, to better control the reduction of Ag cations within the oxide matrix (Ag/Ti:Si/Au).^[^ ^253^ ^]^ It was implemented by embedding Ti nanoclusters into a densified amorphous Si layer. Ti was chosen because of its lower standard reduction potential, thermodynamic compatibility with Si, and ability to form alloys with Ag. The integration of Ti atoms in the a‐Si layer created a significant reduction potential difference with Ag, resulting in a higher probability of Ag cation reduction within the a‐Si medium. This approach effectively enhanced the reduction probability of Ag cations, as demonstrated by the Ti 4.8%: a‐Si device representing a situation of low mobility and high Ag reduction probability (*Γ*_red_ Ag). By utilizing the Ti electrons, the recombination probability could be flexibly adjusted by varying the amount of Ti or incorporating other transition metals with different reduction potentials. Ti clusters effectively induced the electrochemical reduction activity of Ag cations and allowed linear potentiation/depression in tandem with a large conductance range (≈244).

Baek et al. suggested using the specific structural and compositional features to help move the ions.^[^ ^251^ ^]^ In LiCoO_2_, the hexagonal lattice structure with alternating layers of lithium and cobalt ions, as well as the shared edge CoO_6_ octahedra forming CoO_2_ slabs, creates an environment conducive to anisotropic diffusion of lithium ions within the grains. This diffusion occurs transversely along the slabs perpendicular to the *c*‐axis. Additionally, the vertical transport of Li ions parallel to the *c*‐axis takes place via grain boundaries, bypassing the need to penetrate the CoO_2_ slabs. Hence, this leads to a reduction in both the distance covered and the energy expended during ion movement. Consequently, ion mobility becomes more manageable, facilitating the implementation of synaptic device traits. Kwon et al. demonstrated a method that utilized nanostructures to regulate the location and growth of the filaments.^[^ ^254^ ^]^ In nanorod (NR)‐based memristors (Pt/HfO_2_ NR/TiN), the concentration of the electric field at the edge of the NR promoted the formation of oxygen vacancies near the NR surface, facilitating the movement of oxygen ions and enhancing conductive filament growth. The reset process was not constrained by the availability of oxygen ions for recombination, resulting in stable repetitive switching without degradation of the HRS. Moreover, the investigation of conductance‐modulation characteristics revealed an improvement in the linearity of the NR memristors. This enhanced linearity was attributed to the unique property of NR memristors, where conductive filaments were localized near the surfaces of the NRs owing to the field concentration at the edge. As depicted in Figure 7C, this localization, combined with the 1D surface growth accompanying longitudinal growth, led to smaller changes in the conductance per input pulse train and contributed to the observed good linearity.

Achieving a linear relationship between the conductance modulation and programming pulse is crucial for emulating biological synapses in memristor‐based neural networks. Linearity allows for precise adjustment of synaptic weights, accurate inference of input signal intensity, and compatibility with existing algorithms and learning rules. Various approaches have been explored to achieve linearity, including controlling the generation path of conductive filaments through interfaces and grain boundaries, doping metals in the electrolyte layer, and utilizing nanostructures. These findings contribute to the advancement of memristor technologies for achieving more efficient and biologically inspired neural networks.

#### 3.3.3 Synaptic plasticity

**FIGURE 8:**
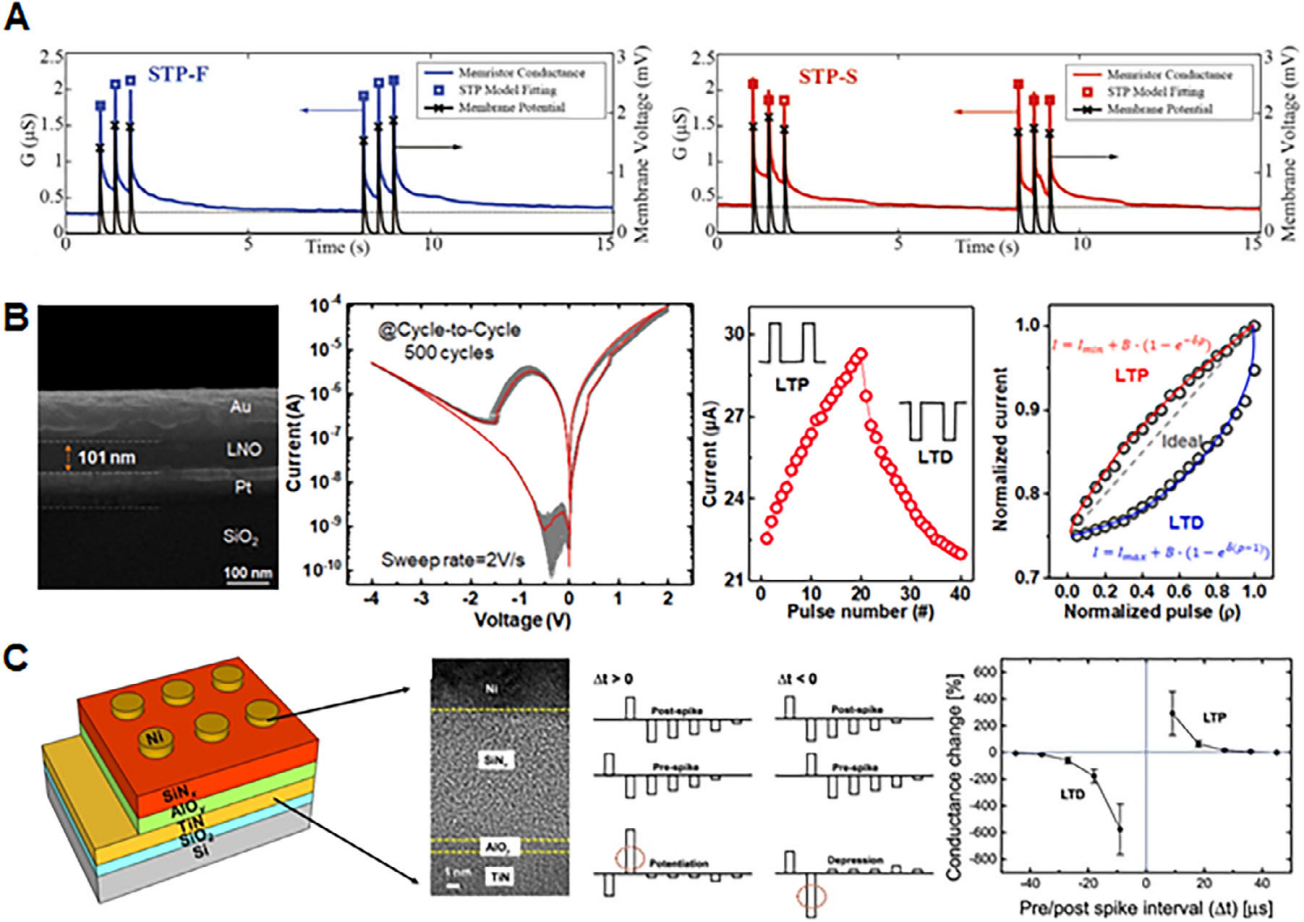
(A) Repeated short‐term plasticity (STP) postsynaptic response with simulations of the contributions of each pulse to a presynaptic neuron membrane potential and appropriate STP model fitting. Reproduced under the terms of the Creative Commons CC BY license.^[^ ^255^ ^]^ Copyright 2016, The Authors, published by Springer Nature. (B) Scanning electron microscopic image of the cross‐section of the Au/LNO/Pt device. (left) 500 cycles of current–voltage (*I*–*V*) curves for cycle‐to‐cycle tests. The median cycle is drawn in red (middle‐left). Long‐term potentiation (LTP) and long‐term depression (LTD) with 20 positive pulses and negative pulses, respectively (middle‐right). Non‐linearity of LTP and LTD with the ideal linearity plotted in a dotted line (right). Reproduced with permission.^[^ ^256^ ^]^ Copyright 2022, Elsevier. (C) Schematic of the Ni/SiN*_x_*/AlO*_y_*/TiN memristive device with a transmission electron microscopic image (left). Systematically designed consecutive pulse trains with different voltage amplitudes (middle). Spike‐timing–dependent plasticity characteristics of the device (right). Reproduced with permission.^[^ ^258^ ^]^ Copyright 2017, American Chemical Society.

Synaptic plasticity involves changes in the strength, efficiency, and structures of synapses, allowing for the modification and encoding of new information. Therefore, mimicking various forms of synaptic plasticity such as LTP, STP, and STDP, is essential for the development and subsequent functioning of artificial synapses. STP is characterized by rapid response and information filtering in synaptic strength, whereas LTP is associated with memory capacity and dynamic changes in synaptic strength. Researchers have successfully implemented STP and LTP using memristors. Berdan et al. demonstrated the STP characteristics in the Pt/TiO_2_/Pt memristor (Figure 8A).^[^ ^255^ ^]^ In order to change the resistance of the memristor, it is necessary to exceed an energy barrier *E*_i_, which enables toggling between different thermodynamic states. The previous state of the memristor influences the specific activation energy *E*_i_ needed for transitioning between states. If the provided energy is insufficient to surpass *E*_i_, a temporary and volatile response is generated, causing a momentary deviation from the initial equilibrium state. Subsequent voltage pulses result in nonuniform modulation of the effective resistance, with the barrier either decreasing or increasing depending on the transition direction. This transient response bears similarities to the reversible modifications observed in synaptic connections, which are referred to as STP.

Wang et al. demonstrated the LTP characteristics using an Ar‐irradiated Au/LNO/Pt memristor (Figure 8B).^[^ ^256^ ^]^ They emulated the working principle of a biological synapse according to various electrical pulses. In this study, the long‐term potentiation and depression characteristics were emulated with identical positive and negative pulses (*VA* = ± 4 V, Δ*t* = 40 ms, *Tw* = 50 ms), producing an impulse sequence consisting of 20 positive pulses and 20 negative pulses. Their results showed that the proposed electronic synapse could realize monotonically increasing or decreasing current levels (corresponding to the device conductance state) with identical voltage pulses. Luo et al. researched the emulation of biological synapses using two‐dimensional WSe_2_ nanosheets as a memristor.^[^ ^257^ ^]^ They exhibited the LTP functionality of controlling and retaining the synaptic weight by applying large‐amplitude voltage pulses that exceeded the SET voltage. Voltage pulses applied to the pre‐synapse or post‐synapse produced long‐term potentiation or long‐term depression, respectively. Because the memristor had a two‐terminal structure, each terminal could act as a pre‐ and post‐synapse, and this simple structure made it easy to control the potentiation and depression of the synapse. Using bipolar non‐volatile behaviours of the WSe_2_ memristor, the research group was able to emulate the biological LTP process successfully.

STDP is a specific synaptic plasticity form based on the precise timing of presynaptic and postsynaptic spikes. It refers to the ability of synapses to change their strengths based on the relative timings of these spikes. In STDP, the timing relationship between the pre‐ and postsynaptic spikes determines whether the synaptic connection is potentiated (strengthened) or depressed (weakened). Kim et al. researched the application of memristors based on the Ni/SiN*_x_*/AlO*_y_*/TiN memristive device.^[^ ^258^ ^]^ The memristor enabled a progressive change in conductance by facilitating the gradual growth and dissolution of the conducting path. The presence of an AlO*_y_* layer in the device enhanced the analog switching performance by reducing the current overshoot by acting as a series resistance. The smooth and continuous switching behaviour made it well‐suited for showcasing synaptic characteristics. These well‐defined characteristics made it feasible to implement STDP. During STDP, when both pre‐ and post‐neuron spikes arrived at the memristor with a delay (*D*_t_), their combined waveform (*V*_pre_—*V*_post_) transiently exceeded the threshold voltage. This led to either an increase (Δ*G* > 0, synapse strengthening) or a decrease (Δ*G* < 0, synapse weakening) in the conductance (*G*) of the memristor, depending on the sign of *D*_t_. Experimental observations confirmed that conductance changes occurred only with closely timed spikes, while long delays did not induce any modification in the device behaviour. Overall, as shown in Figure 8C, the study demonstrated the potential of memristors for implementing STDP, facilitating synaptic plasticity in electronic systems.

## 4 MEMRISTIVE DEVICES IN PARALLEL NETWORKS OF ARTIFICIAL SENSORY SYSTEMS

### 4.1 Detection of external information by memristor‐based sensory system

**FIGURE 9:**
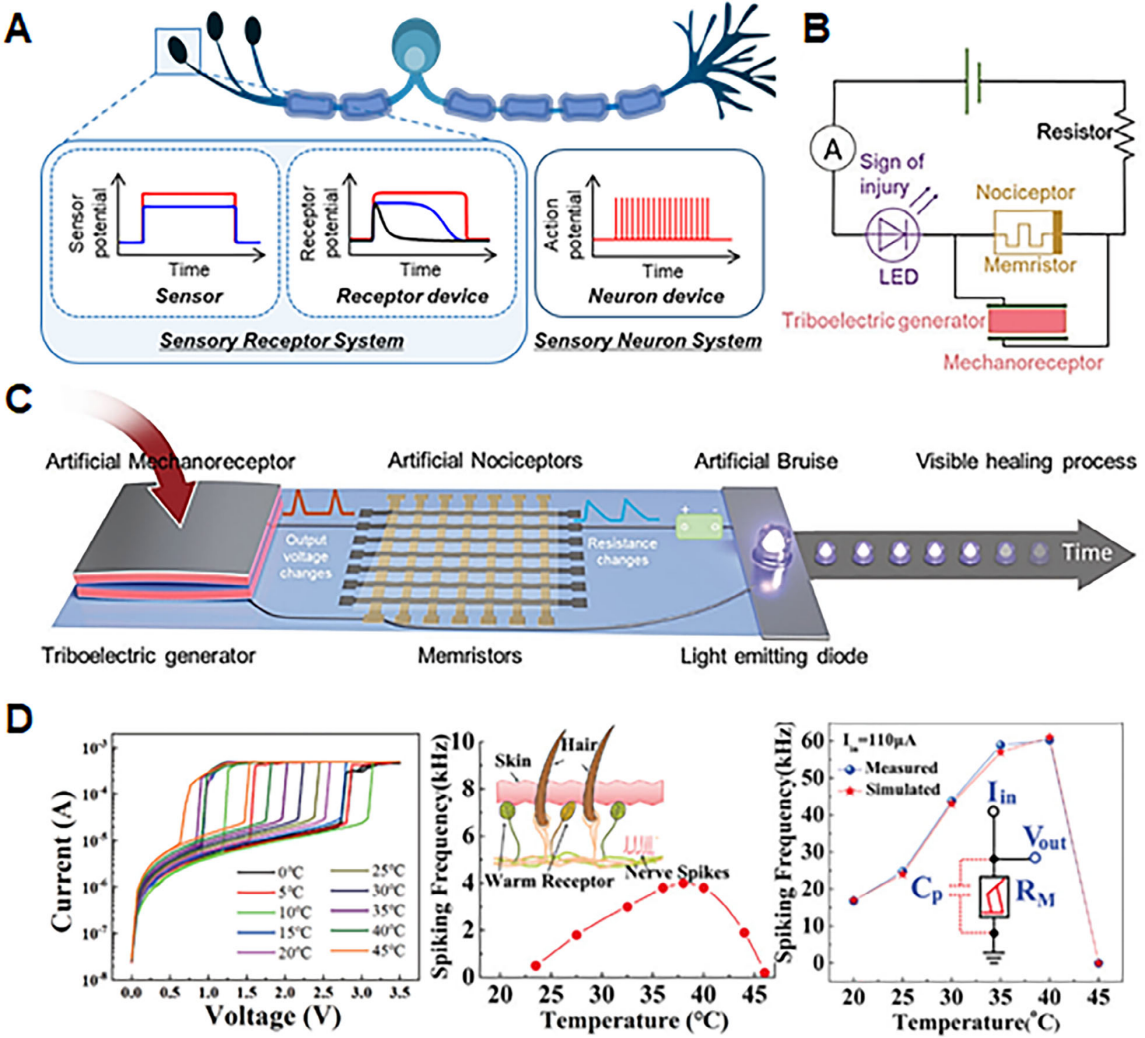
(A) Schematic of sensory receptor system consisting of sensor and receptor parts. (B) Circuit diagram of the corresponding artificial injury response system. Reproduced under the terms of the Creative Commons CC BY license.^[^ ^130^ ^]^ Copyright 2022, The Authors, published by Wiley‐VCH GmbH. (C) Schematic of artificial injury response system to emulate a sense of pain, sign of injury, and healing, based on the integration of a triboelectric generator, memristor receptor device, and light‐emitting diode. Reproduced under the terms of the Creative Commons CC BY license.^[^ ^130^ ^]^ Copyright 2022, The Authors, published by Wiley‐VCH GmbH. (D) Current–voltage (*I–V*) characteristics of the VO_2_ memristor under different stimulus temperatures (left). Schematic illustration and spike‐frequency change of the biological warm receptor under temperature stimulus (middle). Schematic graph of the relationship between the spike frequency and temperature change of the artificial warm receptor. The inset diagram represents the artificial warm receptor circuit (right). Reproduced with permission.^[^ ^274^ ^]^ Copyright 2022, Wiley‐VCH GmbH.

In this section, we introduce the research conducted on the detection and processing of specific external stimuli by establishing an artificial sensory system using the memristor‐based sensory elements introduced in the previous sections. As shown in Figure 9A, a memristor‐based sensory receptor system that generates a continuous form of receptor potential according to the external stimulus consists of sensing and processing parts. The sensor part detects the external stimulus and converts it into an electrical signal, and the receptor device produces a threshold, sensitization, and adaptation reactions according to the applied electrical stimulus. In the following sections, we first introduce studies that sensor‐integrated receptor system connecting sensing parts such as thermoelectric and triboelectric modules, which can convert external analog stimuli into electrical signals. Then, we introduce studies that construct receptor systems with a single receptor device without adding a sensor module. Finally, we present a sensory neuron system that generates spike‐based outputs such as action potentials, according to external stimuli, rather than continuous signals, by configuring a neural circuit using circuit elements such as resistors and capacitors.

#### 4.1.1 Sensor‐integrated receptor system

Most memristor‐based artificial sensory receptor systems are designed by integrating them with various sensors, which can detect specific stimuli and convert them into electrical signals. The type of sensor connected is determined according to the external stimuli. In this subsection, we introduce memristor‐based sensory receptor systems, which are integrated with sensors or electric modules, to generate a continuous form of receptor potentials according to specific stimuli. First, mechanoreceptors, which are specialized sensory receptors that sense mechanical changes such as pressure, vibration, stretching, and contact, are essential to perceiving the physical forces acting on the body.^[^ ^259^, ^260^ ^]^ They play a key role in tactile sensation and proprioception, which is the awareness of the position and movement of the body.^[^ ^261^, ^262^ ^]^ To implement a mechanoreceptor system, various volatile memristive devices have been proposed, which integrate physical sensors such as resistive pressure sensors,^[^ ^263^, ^264^ ^]^ piezoelectric sensors,^[^ ^18^, ^59^ ^]^ and triboelectric nanogenerators (TENGs).^[^ ^130^, ^265^ ^]^ For example, by integrating a self‐powered TENG device to a threshold‐switching memristor (Ag/h‐BN/Ag) through a rectifier bridge circuit, Ding et al.^[^ ^266^ ^]^ demonstrated a mechano‐nociceptor system that could mimic the signature nociceptor features (threshold, relaxation, allodynia, etc.). The connected TENG was exploited to sense the external mechanical stimulus and provide intensity‐dependent electrical signals to the memristive devices.

In addition, for emulating the functions of the reflex arc, which is an important phenomenon that enables rapid reactions in response to harmful stimulus, He et al.^[^ ^264^ ^]^ demonstrated an artificial reflex arc system composed of three primary elements involving a pressure sensor, mechanoreceptor device (Ag/zeolitic imidazolate framework‐8 (ZIF‐8)/Au), and electrochemical actuator. The ZIF‐8‐based mechanoreceptor device was activated at a pressure exceeding a threshold, triggering the electrochemical actuators to complete the motion. This response mechanism closely emulated the all‐or‐none principle observed in the human nervous system. Furthermore, to implement the human‐like injury response system, Xu et al. demonstrated a bioinspired injury response system capable of sensing pain, showing signs of injury and healing (Figure 9B,C). They fabricated an artificial mechano‐nociceptor device based on a copolymer of chlorotrifluoroethylene and vinylidene fluoride (FK‐800)^[^ ^130^ ^]^ (Ag/FK‐800/Pt), demonstrating the four signature nociceptive characteristics. By integrating a triboelectric generator, mechano‐nociceptor device, and light‐emitting diode, the somatic self‐protective modality under noxious stimuli was implemented.

Memristor‐based thermoreceptor devices, which are specialized sensory receptors that respond to temperature changes,^[^ ^267^, ^268^ ^]^ were created by connecting with a thermoelectric module, which could convert a temperature gradient into electrical power.^[^ ^74^, ^128^ ^]^ For example, Song et al.^[^ ^74^ ^]^ mimicked an artificial thermoreceptor using the adaptive and maladaptive receptor devices mentioned in the previous section. As humans detect temperatures of 15−45°C as harmless,^[^ ^269^ ^]^ and those beyond this region as harmful, the experimental temperatures were set at 40°C, 70°C, and 90°C to replicate the harmless and harmful stimuli, respectively. When the device was under the harmless temperature of 40°C, only the rapid and slow‐adapting receptors reacted and adapted to the external voltage stimulus. On the other hand, when the device was under the noxious temperature of 70°C, the maladapting receptor reacted and remained at an onset status to the stimulus. These behaviours successfully mimicked the selective response of biological thermoreceptors.

#### 4.1.2 Single‐device receptor system

A single‐device receptor system is implemented as a single device without integrating a sensor module, using a stimulus‐detectable material in a memristive device. Some attempts have been made based on pressure‐^[^ ^270^, ^271^ ^]^ and light‐responsive^[^ ^272^, ^273^ ^]^ materials; however, so far, the most commonly implemented form is that of temperature receptors designed to respond to temperature changes.^[^ ^274^, ^275^, ^276^ ^]^ For example, memristors have shown potential for acting as thermoreceptors by incorporating thermosensitive materials into the insulating layer. Han et al. demonstrated an artificial warm receptor with VO_2_ Mott memristors (Pt/VO_2_/Pt) (Figure 9D).^[^ ^274^ ^]^ VO_2_, known for its insulator–metal transition at a low temperature of 68°C, served as a key component in these memristors. The VO_2_ memristor could function as a standalone thermal sensor, exhibiting a decrease in the threshold voltage as the temperature increased, enabling it to sense and respond to changes in temperature. They focused on developing a single‐device artificial thermoreceptor and successfully emulated the human skin properties by fabricating the device on a flexible polyethylene naphthalate substrate. Using this thermal sensitivity of VO_2_, Duan et al.^[^ ^275^ ^]^ demonstrated a thermoreceptor system with a VO_2_‐based memristive device (Au/Ti/VO_2_/Ti/Au). Through the serial connection of a piezoresistive sensor and VO_2_‐based thermoreceptor device, a multimodal haptic/temperature receptor system was demonstrated, which could detect and encode pressure and temperature inputs based on the voltage‐dividing effect and intrinsic thermal sensitivity of VO_2_. Shi et al.^[^ ^276^ ^]^ demonstrated an artificial thermal nociceptor system based on bismuth selenide (Bi_2_Se_3_), which is a thermoelectric film facilitating in‐situ temperature sensing (Ag/Bi_2_Se_3_/PMMA/ITO). Combined with a robotic manipulator, it could demonstrate the nerve reflex action under thermal stimulation. By conducting temperature‐dependent tests utilizing the excellent thermoelectric conversion performance of Bi_2_Se_3_, the neuractivity of human heat receptors under high‐temperature stimulation was well emulated. Table 3 presents the summary of the function and switching characteristics of different receptors.

**TABLE 3:** Summary of artificial receptor that implement the function of the sensory system.

Sensory system	Role	Structure	Operating voltage	Output range	Sensor	Ref
Nociceptor	Sensing the potentially harmful or damaging stimuli, transmitting signals associated with pain perception to the central nervous system.	Pt/HfO_2_/TiN	4 V	0–3 μA	–	^[^ ^79^ ^]^
		Pt/TiO_2_ Nanobelt/Pt	20 V	0–0.4 μA	–	^[^ ^276^ ^]^
		Au/MoS_2_/Ag	0.25 V	0–125 μA	–	^[^ ^277^ ^]^
		Ag/ZrO_x_/Pt	0.4 V	0–10^‐4^ μA	–	^[^ ^278^ ^]^
Mechanoreceptors	Sensing mechanical changes such as pressure, vibration, stretch, and touch	Pt/VO_2_/Pt	0.6 V	<130 μA	–	^[^ ^274^ ^]^
		Ag/h‐BN/Ag	1.2 V	0–40 μA	Triboelectric generators	^[^ ^266^ ^]^
		Ag/FK‐800/Pt	3 V	200–500 μA	Triboelectric generator	^[^ ^130^ ^]^
Thermoreceptors	Sensing the temperature changes to protect organisms from extreme temperatures	Pt/Ag/SiO_2_/Ag/Pt	0.5 V	10–20 μA	Thermoelectric module	^[^ ^74^ ^]^
		Pt/VO_2_/Pt	0.6 V	0.3–0.7 V	–	^[^ ^279^ ^]^
		Au/Ti/VO_2_/Ti/Au	1.5 V	1–2 V	–	^[^ ^275^ ^]^
Photoreceptors	Converting light into electrical signals	Au/AZO/PMMA/ITO	4 V	20–60 μA	UV sensor	^[^ ^131^ ^]^

#### 4.1.3 Spike‐based sensory neuron system

To efficiently process vast amounts of data, a memristor neuron must convert the transmitted signals generated by the receptor system to spike signals (action potentials). In this subsection, we sequentially introduce spike‐generating artificial sensory neuron systems corresponding to three human sensory systems—tactile, visual, and olfactory sensory systems.

**FIGURE 10:**
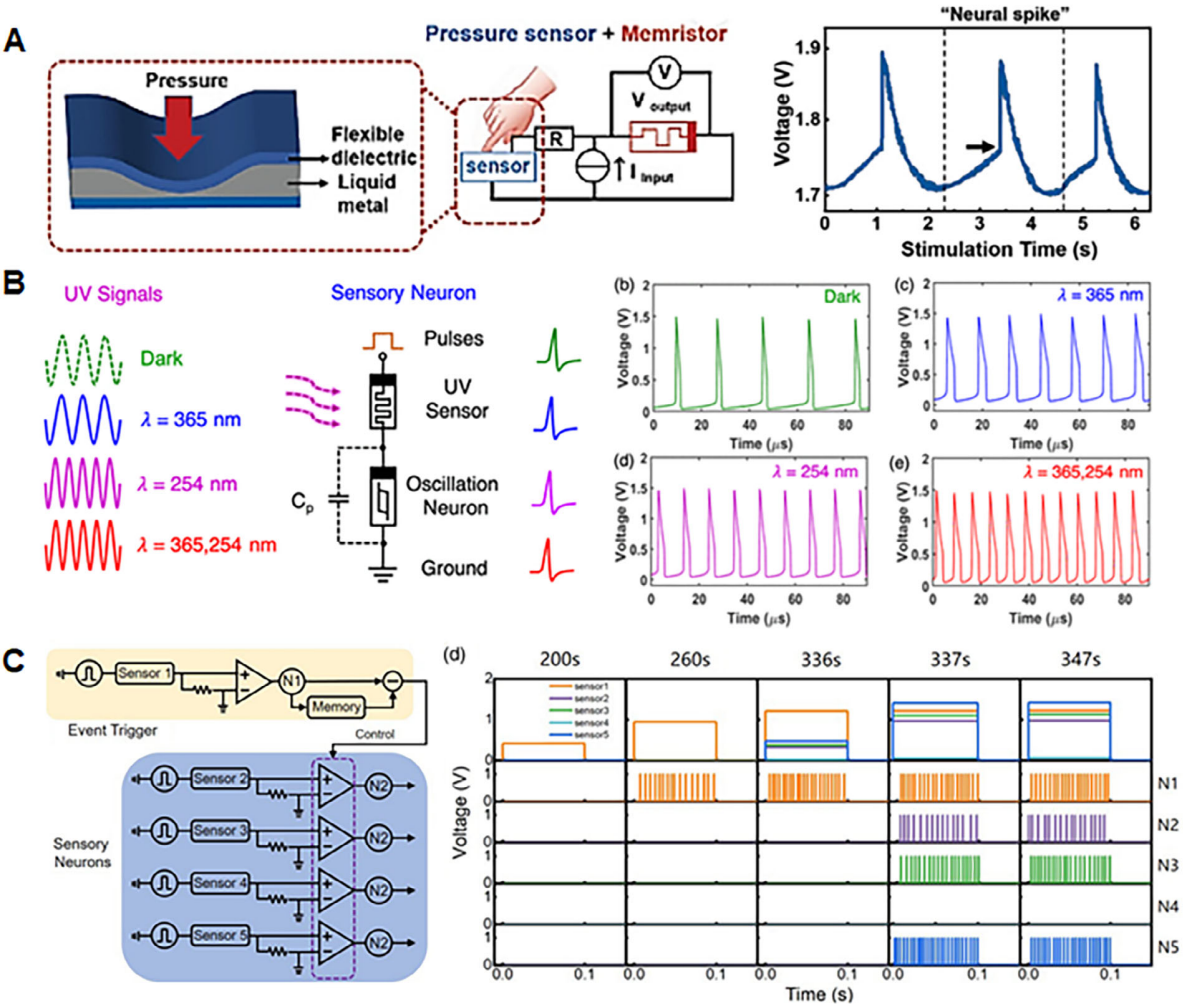
(A) Circuit diagram of the Pt/Co_3_O_4−_ *_x_*/ITO memristor‐based artificial tactile sensory neuron, comprising a pressure sensor, resistive pressure sensor, Pt/Co_3_O_4−_ *_x_*/ITO memristor, and constant‐current source. Reproduced with permission.^[^ ^280^ ^]^ Copyright 2022, Wiley‐VCH GmbH. (B) Schematic diagram of the ultraviolet (UV) signals of different wavelengths and the circuit of the artificial sensory neurons (left). Voltage–time graph representing the voltage spikes generated by the neuron under different UV signals (right). Reproduced with permission.^[^ ^284^ ^]^ Copyright 2020, American Chemical Society. (C) Schematic diagram of the circuit of the event trigger and sensory neurons (left). Output spikes generated by the sensory neurons in response to input signals, for toluene. The first row represents the input voltages and the rest of the rows below are the output spikes from the first to the fifth sensory neuron respectively (right). Reproduced under the terms of the Creative Commons CC BY license.^[^ ^21^ ^]^ Copyright 2022, The Authors, published by Wiley‐VCH GmbH.

The tactile sensory neuron encodes stimulus by translating external mechanical stimulus into modulated electrical spikes. Li et al. demonstrated a spike‐generating NbO*_x_*‐based neuron system (Ta/NbO*_x_*/Pt) by integrating a polypyrrole(PPy)‐based micropyramidal resistive pressure sensor.^[^ ^263^ ^]^ The NbO*_x_*‐based memristor could generate the spikes when connected in series with a load resistor. The micropyramidal structures in the PPy films enabled high sensitivity in detecting low pressure levels, making them suitable for wearable electronic‐skin applications. The pressure‐sensing mechanism relied on the deformation of these structures, which changed the contact area between the PPy and interdigitated Au electrodes, resulting in variations in resistance. This setup allowed for an event‐driven approach, where external pressure triggered the activation of the artificial mechanoreceptor system. When no or insufficient pressure was applied, the mechanoreceptor remained in an “OFF” state. Electrical spiking was initiated once the applied pressure reached the threshold (≈390 Pa, similar to a gentle touch). The frequency of these electrical spikes was positively correlated with the applied pressure, mimicking the modulation of spiking frequencies observed in biological neural responses. When the pressure exceeded a certain threshold (>700 Pa), no spikes were generated, replicating the behaviour of biological mechanoreceptors faced with strong stimuli. Xie et al. also reported a tactile sensory neuron system using a Pt/Co_3_O_4‐x_/ITO‐based ionic memristor with a pressure sensor connected in parallel (Figure 10A).^[^ ^280^ ^]^ The memristor exhibited adaptive behaviour when continuous innocuous stimuli were applied, because of the strengthening of the conductive filament formed in the memristor. Therefore, when consistent pressure at the same amplitude, *F*_a_, was applied to the pressure sensor, the memristor generated spikes whose frequency decreased over time. Because of this neuron system's integration and firing capabilities, an unambiguous pressure route could be easily extracted by suppressing or significantly increasing the input signal.^[^ ^275^ ^]^

Visual sensory neurons in the retina receive optical signals and transform them into electrical spike signals. Pei et al.^[^ ^281^ ^]^ developed a fully‐memristor‐based artificial visual perception nervous system that consisted of a quantum‐dot (QD)‐based optoelectronic memristor (TiN/PbS QD/ITO) and a MoS_2_ nanosheet‐based threshold‐switching memristor (Ag/MoS_2_ nanosheet/Ag/MoO*_x_*/Ag).^[^ ^282^ ^]^ In this device configuration, PbS QDs orderly assembled themselves on an ITO electrode and displayed a multiband fluorescence impact upon exposure to light, allowing for the detection and processing of photoelectric signals. In its pristine state, the device showed resistance changes under light illumination. Pei et al. constructed a circuit for an artificial visual perception nervous system based on this optoelectronic memristor. When light was illuminated, the system created a higher amplitude and greater frequency of spikes because of the resistance change in the memristor. Moreover, Pei et al. suggested a highly efficient artificial visual sensory system consisting of an optoelectronic threshold‐switching memristor and actuator.^[^ ^283^ ^]^ In this work, an Sb_2_Se_3_/CdS‐core (SC)‐based memristor array enhanced the light‐harvesting activities, received optical signals, and converted them to a voltage before transmitting them to the threshold‐switching memristor‐based neuron circuit. When the SC devices were not exposed to light, there was a slight resistive‐switching phenomenon, which was not prominent. However, the resistive‐switching effect was significantly enhanced when light was applied. The response current increased and the resistance ratio between the ON and OFF states improved. The mechanism behind the increase in the response was the photosensitive behaviour of the SC material. When illuminated by light, electron–hole pairs were generated. The photogenerated holes were trapped in the trapping sites of Se_2_Sb, and the photogenerated electrons were the results of photoconductive gain. When the optoelectronic neuron memristor was activated, it could cause an electrochemical actuator to move, emulating eye‐muscle contraction and reproducing the self‐protection behaviour of closing the eyes when exposed to intense light.

Similarly, to implement UV sensory neurons detecting UV damage, Wu et al.^[^ ^284^ ^]^ demonstrated a simple artificial visual sensory neuron composed of an IGZO_4_‐based ultraviolet (UV) sensor and NbO*_x_*‐based memristor neuron (Figure 10B). When UV light was illuminated, the optical signals were converted to electrical signals in the form of a photocurrent (*I*_photo_) via an IGZO_4_‐based UV sensor. The photo from the UV sensor served as an optoelectronic converter and was transmitted to the next NbO*_x_*‐based memristor neuron to generate spike signals. As the wavelength decreased, the resistance of the UV sensor decreased, resulting in a corresponding change in the resistance of the memristor element in response to the voltage applied. Therefore, the hybrid configuration of a photodetector that received optical input and a neuron that generated electrical spike signals produced the light‐modulated neuronal characteristic that the frequency was increased by light illumination. However, this hybrid sensory neuron could detect only UV signals because of the UV sensor's limited light‐absorption ability.

Olfactory neurons detect odorants from the environment and generate electrical spikes. To implement the olfactory sensory neuron system, Wang et al. demonstrated a LIF olfactory sensory neuron with a volatile memristive device (Pt/Ag/TaO*_x_*/Pt) integrated with an array of gas sensors to convert the chemical information of gases, such as toluene, into electrical spikes (Figure 10C).^[^ ^21^ ^]^ The signal was transmitted from the LIF sensory neurons to the relay neurons through a non‐volatile memristor synapse device (Pt/Ta/TaO*_x_*/Pt), according to the synaptic weight. After learning and training with the supervised spike‐rate‐dependent plasticity, the gas features were memorized in the memristive device conductance, and eventually, the relay neurons classified gases with different firing patterns.

### 4.2 Integration and processing of spikes signal by memristor‐based neural network system

The synapse, which is the connection between neurons that allow the communication and transmission of signals in the brain, plays a crucial role in learning and information processing. In reinforcement learning, synapses play a fundamental role in forming and strengthening associations between actions and rewards.^[^ ^285^, ^286^, ^287^, ^288^ ^]^ When an individual receives positive reinforcement or a reward for a certain behaviour, the synapses involved in the neural pathways related to that behaviour are strengthened (synaptic plasticity). This synaptic plasticity enables the brain to learn and optimize decision‐making processes based on the outcomes of the previous actions. Through repeated reinforcement and synaptic modification, the brain can develop strategies and preferences that maximize rewards and minimize negative consequences. Furthermore, perception refers to how organisms interpret and make sense of sensory information from the environment.^[^ ^285^, ^289^, ^290^, ^291^ ^]^ It involves collecting, organizing, and interpreting sensory data to construct a meaningful representation of the world. Perception is a complex cognitive process in the brain, involving sensory inputs from various modalities such as vision, hearing, touch, taste, and smell. These sensory inputs are transmitted through specialized sensory receptors, which convert physical stimuli into electrical signals that the brain can process.

Inspired by the human brain, the neuromorphic computing paradigm offers an intriguing approach for future computing systems. Unlike traditional architectures, the architecture of the brain is massively parallel, with numerous interconnected low‐power computing elements (neurons) and adaptive memory elements (synapses). This parallelism facilitates vector matrix multiplication (VMM), a fundamental operation in linear algebra and mathematical computations,^[^ ^292^, ^293^, ^294^, ^295^ ^]^ which underlies many AI algorithms and enables them to process and learn from data, making it an essential component in AI research, development, and applications. VMM can be implemented by adjusting and inputting weights related to connections between nodes or neurons. In this section, we will provide an overview of the various types of neural‐network algorithms and the available research on the implementation of each neural network as an artificial neuron and synapse, facilitating the performance of human‐like tasks such as recognition, classification, and memory.

#### 4.2.1 ANN and DNN and SNN

**FIGURE 11:**
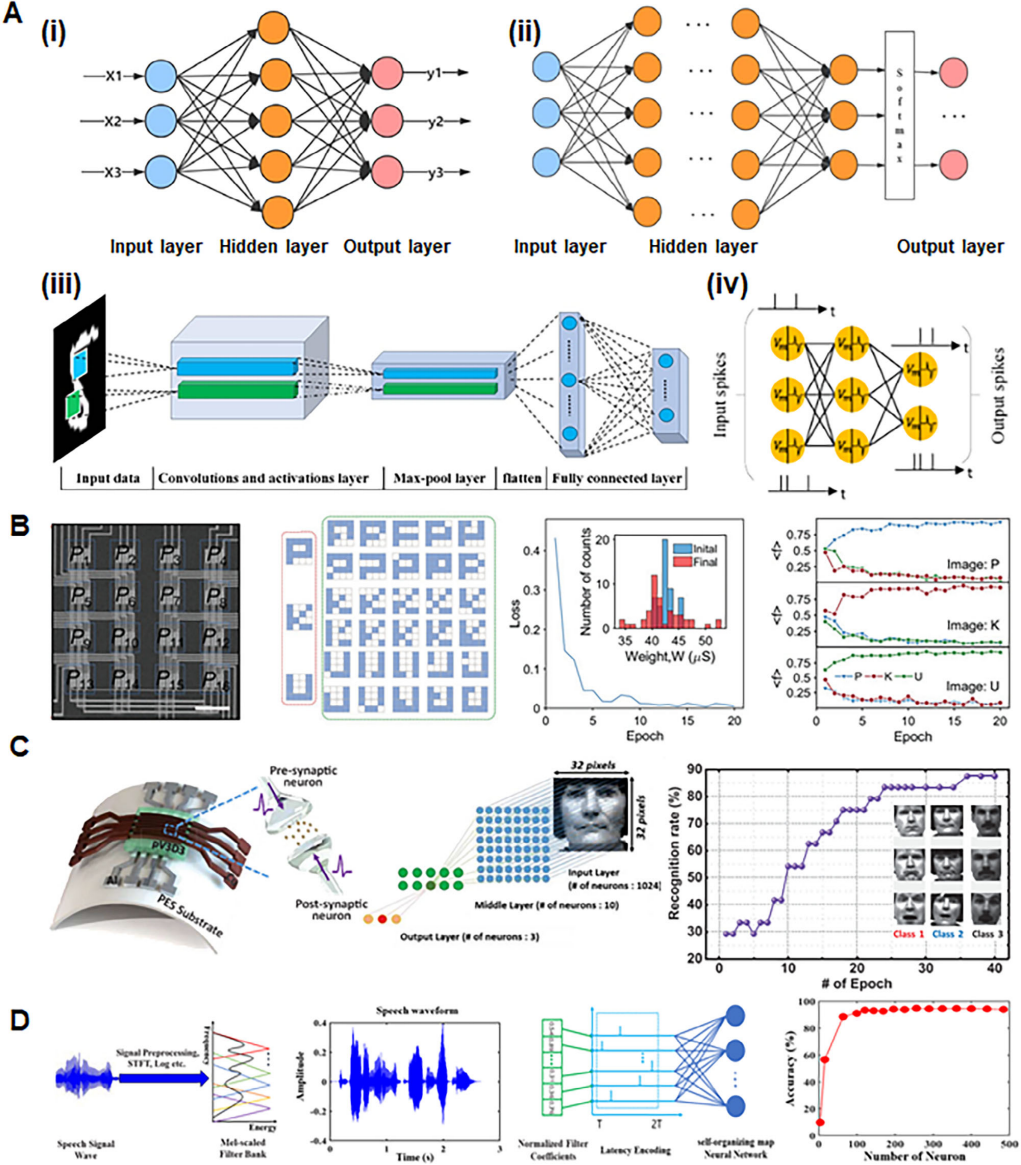
(A) Schematic of the neural networks: artificial neural network (ANN), deep neural network (DNN), Convolution neural network (CNN) and spiking neural network (SNN). Reproduced with permission.^[^ ^322^ ^]^ Copyright 2019, Elsevier, Reproduced under the terms of the Creative Commons CC BY license.^[^ ^323^ ^]^ Copyright 2021, The Authors, published by Frontiers Media S.A. Reproduced under the terms of the Creative Commons CC BY license.^[^ ^324^ ^]^ Copyright 2022, The Authors, published by MDPI. (B) Scanning electron microscopic image of the 16 × 3 1Phototransistor‐1memristor array with 4 × 4 pixels included (left). Training and test set of the letters “P,” “K,” and “U” (middle‐left). Loss function during 20 epochs; inset represents the weight distribution of the initial and final states of the array (middle‐right). The activation function output of three classifications (right). Reproduced with permission.^[^ ^309^ ^]^ Copyright 2022, Wiley‐VCH GmbH. (C) Schematic illustration of the flexible pV_3_D memristor mimicking the biological synapse (left). pV_3_D_3_‐based ANN for face classification of 32 × 32 grayscale image (middle). Recognition rate after a number of training epochs. The inset shows nine training images of three people making three different facial expressions, extracted from the Yale Face Database (right). Reproduced with permission.^[^ ^320^ ^]^ Copyright 2019, American Chemical Society. (D) Schematic diagram of extracting and preprocessing the speech‐signal waveform in order to generate compressed feature representation (left). Example of the waveform of the speech signal (middle‐left). Schematic diagram of the self‐organizing map (SOM) neural network (middle‐right). Schematic graph showing the accuracy changes with the increase in the number of neurons used in the SOM (right). Reproduced under the terms of the Creative Commons CC BY license.^[^ ^314^ ^]^ Copyright 2021, The Authors, published by Wiley‐VCH GmbH.

The artificial neural network (ANN) is a machine‐learning technology designed to replicate and understand human capabilities by simulating biological neural networks.^[^ ^296^, ^297^, ^298^, ^299^ ^]^ ANN operates through interconnected neurons, each representing a specific output function known as a stimulus function. The connections between neurons, referred to as weights, store information within the ANN (Figure 11A‐i). The output of the network depends on the network connectivity, weight values, and incentive functions. ANN hardware is based on a memristor crossbar, which can be achieved by integrating with peripheral neuron circuits. VMM, a core computing operation associated with the memristor crossbar, can be implemented in dense crossbar geometries using the Ohm's law and Kirchhoff's current law for summation.

Furthermore, deep neural networks (DNNs) (Figure 11A‐ii), also known as deep learning, is a machine‐learning technique that focuses on learning data representations. It is a method inspired by the structure and functioning of human neural networks. Deep learning builds upon the concept of ANNs and has evolved from the previous ANN models. It encompasses various types of networks, including convolutional neural networks (CNNs),^[^ ^300^ ^]^ recurrent neural networks (RNNs),^[^ ^301^, ^302^ ^]^ and generative adversarial networks (GANs)^[^ ^303^, ^304^, ^305^ ^]^ that have emerged in the recent years. Each type has its own specialization—CNNs excel in image processing and recognition, RNNs effectively handle sound and time‐series data, and GANs are proficient in generating images. Although memristor‐based GANs are still in the simulation stage, they hold promise for future development.

CNN, one of the DNNs, is a type of feed‐forward neural network that includes convolutional and pooling layers (Figure 11A‐iii). The CNN architecture comprises an initial input layer, convolutional layer, activation function, pooling layer, and fully connected (FC) layer. Spatial hierarchies from the input data involve details concerning the spatial connections, structures, and layouts of different components within the image. For example, in an image of a face, spatial features could include the arrangement of eyes, nose, and mouth, the relative distances between these features, and the angles at which they are positioned. With in a CNN, the convolutional layer consists of several sets of convolutional filters (also referred to as kernels), which possess the capability to grasp spatial hierarchies of attributes from the provided input images. The quantity and dimensions of these convolution kernels are contingent upon the input data. In CNN operation, it is performed by sliding a kernel continuously over the input blocks generated by the input layer and calculating the sum of weights between the shared local kernel and the input blocks. Then, it moves through the activation and pooling layers to decipher intricate connections, diminishes data values to alleviate computational load, manages overfitting, and conveys the computed outcome to the FC layer. FC layer connects every neuron from the previous layer to every neuron in the subsequent layer. The FC layers at the end of the CNN process the extracted features and make final predictions based on the learned features. CNN can be shared during data processing, facilitating efficient computations while conserving the spatial arrangement of the input image. Moreover, due to weight sharing in convolutional layers, it requires fewer parameters compared to fully connected networks, making them computationally efficient for image processing tasks.

The SNN represents a third‐generation neural network paradigm that aims to process data in a biologically inspired manner (Figure 11A‐iv). Unlike current ANNs, which lack biological accuracy, SNNs bridge the gap between neuroscience and machine learning by utilizing models that closely resemble the actual neuronal mechanisms.^[^ ^306^, ^307^ ^]^ In an SNN, neurons serve as fundamental units responsible for receiving, integrating, and transmitting information in the form of discrete electrical pulses called spikes or action potentials. The information is first translated into pulses with timing and frequency, in the SNN. Correspondingly, the output signals are encoded using timing and frequency, allowing for the representation of the information through precise timing and spiking patterns. Several spiking neuron models with biological characteristics, such as the Hodgkin–Huxley (HH) model, LIF model, and spike response model (SRM), have been developed to simulate real neuron behaviour. Within SNNs, STDP is a synaptic plasticity rule that governs the adjustment of synaptic strengths based on the precise timing of presynaptic and postsynaptic spikes. STDP enables the SNN to adapt and learn from its input patterns, enhancing the network's ability to recognize temporal patterns, perform associative learning, and encode information efficiently. By combining the spiking behaviours of neurons and the STDP of synaptic plasticity, SNNs can process and learn from spatiotemporal information in a manner that closely resembles the functioning of the human brain. Through their ability to process data in spatiotemporal domains and their energy‐efficient operation, SNNs have the potential to advance the field of neural networks and find applications in various domains ranging from artificial intelligence to robotics. The neuronal units in SNNs are only active when receiving or sending spikes, significantly reducing the energy consumption. This event‐driven behaviour allows for energy saving and makes SNNs well‐suited for low‐power applications.

#### 4.2.2 Pattern recognition

In neuromorphic systems, neural networks are employed to perform pattern‐recognition tasks. They consist of interconnected artificial neurons and synapses, which process and transmit information similar to biological neurons and synapses. When an external stimulus is applied to an artificial neuron, the neuron generates a spike signal, which is applied to the synaptic memristor array to modulate the synaptic weights. Through this process, the network undergoes iterative weight adjustment of its connections in response to input patterns and their associated labels, enabling it to learn. These adjustments in synaptic weights train the neural network to recognize and classify patterns. Neuromorphic computing is more advantageous for pattern recognition, than the traditional computing approaches. Its parallel‐processing architecture and distributed memory storage enable efficient and rapid large‐scale data processing, allowing for pattern recognition in various applications.

Peng et al. focused on pattern recognition using synapse crossbar arrays (consisting of TiN/TaO*_x_*/HfO*_x_*/TiN), which are arrays of memristive devices, at each intersection.^[^ ^308^ ^]^ They developed high‐yield and high‐performance memristor crossbar arrays that integrated multiple arrays for improving the parallel‐computing efficiency. They also proposed a hybrid training method to address device imperfections and enhance system performance. A five‐layer memristor‐based CNN was built and tested for image recognition using the MNIST dataset, achieving a high accuracy of over 96%. The system demonstrated parallel convolutions with shared inputs and the replication of multiple kernels in memristor arrays for processing different inputs simultaneously. The memristor‐based CNN showed energy efficiency over two orders of magnitude greater than that of the state‐of‐the‐art graphics‐processing units. It was also scalable to larger networks like residual neural networks. This indicated that a promising hardware solution for deep neural networks and edge computing could provide an efficient and energy‐saving alternative to the traditional von Neumann architecture.

Dang et al. fabricated a 1‐transistor‐1‐memristor (1T1R) array (16 × 3), which was composed of light‐programmable zinc‐oxide thin‐film transistors and Mo/SiO_2_/W memristors with non‐volatile memory properties, and used it to mimic an image perception network (Figure 11B).^[^ ^309^ ^]^ It operated as a single‐layer perceptron and was designed to recognize the letters “P,” “K,” and “U,” in a 4 × 4 pixel image‐recognition task. Randomly chosen letters were optically projected during each training epoch. The training process involved 0.5−0.55 V/ 100 μs word‐line bias voltages to write/erase the 1T1R devices, with 0.5 V bias voltage in each select line. The training results showed that the 1T1R array performed exceptionally well, achieving a recognition accuracy of 99.3% with only ten epochs on a noisy test set. The initial and final weight histograms indicated a transformation from a tight conductance distribution to a wide distribution. The evolution of the three classifier neurons (f1, f2, f3) showed their variations across each epoch. The high‐linearity light‐tunable weight characteristics of the 1T1R device demonstrated excellent performance and facilitated efficient online training.

The combination of novel neural networks and artificial synapses has demonstrated remarkable accuracy in letter‐recognition tasks. These promising outcomes indicate the potential of neuromorphic computing systems for replicating the structure and functionality of the human brain. This represents a significant advancement towards achieving efficient and parallel information processing with minimal power consumption.

#### 4.2.3 Classification and prediction of complex problem

Beyond mere image recognition, neural networks have the capability for higher‐level learning and understanding through network training. By capturing complex patterns and relationships in the data, neuromorphic systems can achieve high accuracy in tasks such as image recognition,^[^ ^310^, ^311^, ^312^ ^]^ speech recognition,^[^ ^15^, ^313^, ^314^ ^]^ natural language processing,^[^ ^315^, ^316^, ^317^ ^]^ and much more.^[^ ^318^, ^319^ ^]^ Furthermore, the energy efficiency of neuromorphic systems, inspired by the brain's low‐power operation, makes them particularly suitable for edge computing and AI applications with limited power resources.

Jang et al. demonstrated the use of poly (1,3,5‐trivinyl‐1,3,5‐trimethyl cyclotrisiloxane) (pV_3_D_3_) memristors as flexible synaptic devices for pattern recognition (Cu/pV_3_D_3_/Al).^[^ ^320^ ^]^ An ANN with a crossbar architecture was employed to classify facial images. The ANN consisted of input, middle, and output neurons, and utilized a two‐crossbar layout for unsupervised learning and classification. The memristor‐based ANN performed VMMs, facilitating essential machine‐learning operations. Training images were provided to input neurons, which emitted presynaptic spikes based on the pixel intensities. Memristor synapses generated postsynaptic currents, which were integrated by output neurons using an LIF circuit. The conductance matrix of the memristor array facilitated direct VMM, producing postsynaptic currents and updating the synaptic weights. A simplified STDP learning rule was employed for weight updates, simplifying the peripheral circuitry. As shown in Figure 11C, the ANN was trained and evaluated using a test set of 24 facial images, achieving an average recognition rate of 88%.

Li et al. demonstrated the use of a Ta/HfO_2_‐based memristor, acting as a long–short‐term memory (LSTM)–RNN for gait‐based human identification.^[^ ^321^ ^]^ Gait, as a biometric feature, is advantageous in long‐range identification scenarios where other biometrics such as the face, may not be useful. The LSTM–RNN memristor utilized a two‐layer architecture implemented with a memristor crossbar array. Feature vectors extracted from video frames were used as input, and the classification result was obtained as electric current. The system was trained and tested using video sequences from different individuals. The training involved minimizing cross‐entropy through root‐mean‐square propagation, and the classification accuracy reached 79.1% after 50 epochs of training. The in‐situ training on the LSTM memristor network demonstrated its effectiveness, and a comparison with a digital counterpart showed comparable accuracy. However, achieving higher accuracy in conductance tuning and improving the crossbar array is necessary for further advancements. The study also highlighted the sensitivity of the LSTM network to weight update errors and the potential for different performance requirements with emerging analog devices.

Speech recognition involves the ability to learn audios that are closely related to a particular event sequence. Although speech recognition is widely implemented in software neural networks, a hardware implementation based on energy‐efficient computing architecture is rarely reported. Wu et al. reported speech‐signal recognition based on SNNs using W/MgO/SiO_2_/Mo memristor arrays with multilevel resistance states, where the weights of the artificial synapses in the memristor array could be tuned precisely by voltage pulses (Figure 11D).^[^ ^314^ ^]^ The process involved encoding analog audio signals into spikes and extracting features using the Mel‐frequency cepstral coefficients. Self‐organizing maps were used to handle a large number of spikes, and the maximum‐margin tempotron algorithm was employed for classification. The SNN was trained using a memristor crossbar and achieved a high accuracy of approximately 94% on the test dataset. The speech‐recognition system demonstrated advantages in terms of simplified structure, fewer iterations, and high energy efficiency, compared to other systems based on ANNs. These findings suggest that artificial synapses have the potential to be utilized in diverse applications such as image and pattern recognition, natural language processing, sensory processing, optimization problems, and decision‐making tasks. These opportunities present several potential benefits in fields such as autonomous driving, medical diagnostics, smart sensors, intelligent robotics, and advanced data analytics.

## 5 CONCLUSIONS AND PERSPECTIVES

Biological sensory systems structurally and functionally interconnect sensory receptors, neurons, and synapses, and demonstrate excellent time and energy efficiencies by simultaneously detecting and processing large amounts of analog environmental data. Many studies have tried to implement artificial sensory systems to emulate the efficiency of biological sensory systems. Memristors are powerful devices with numerous advantages such as low energy consumption, fast response time, and efficient scalability, making it ideal for neuromorphic computing. Over the past few years, remarkable progress has been made in the evolution of memristor‐based artificial sensory systems that skilfully mimic biological sensing behaviour, by utilizing various materials, structures, and mechanisms. For the implementation of an artificial sensory system, it is important to understand the characteristics and performance of each sensory component (receptors, neurons, and synapses), as each performs unique roles and functions in the sensing process.

**FIGURE 12:**
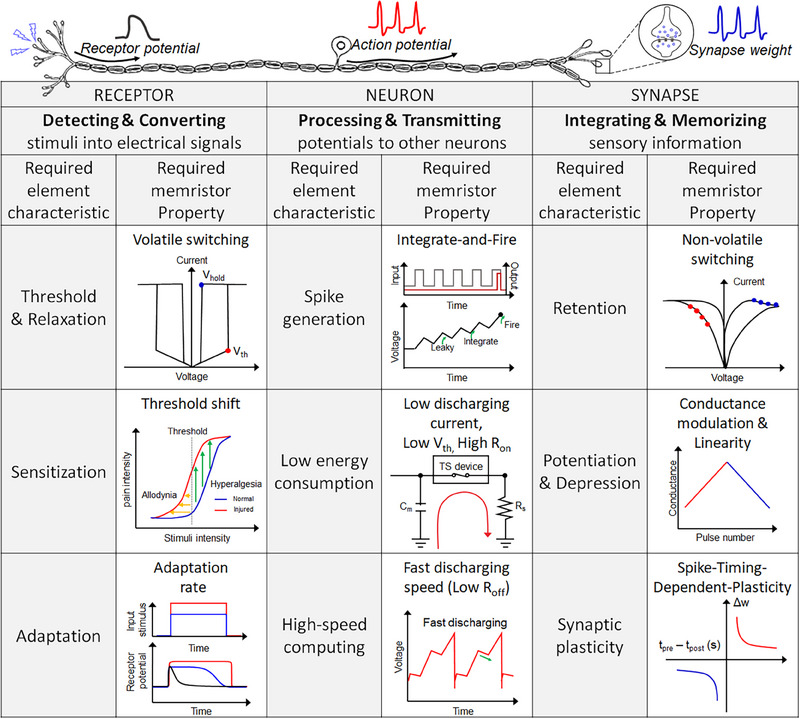
Function and key characteristics of each sensory element (receptor, neuron, and synapse) in a sensory system, and the relevant properties of the memristive devices required to mimic them.

In this review, we suggest the functions and key characteristics of each sensory element (receptors, neurons, synapses) for implementing an artificial sensory system and explain the relevant properties of the memristive devices necessary to implement them (Figure 12). We also classify the reported works on artificial sensory systems based on the three main components. In summary, sensory receptors, which detect analog signals from the external environment and convert them into electrical signals, necessitate threshold and relaxation characteristics. As sensory receptors have a specific threshold, representing the minimum intensity of stimulus required to activate, these can be implemented using memristive devices with volatile switching properties. Sensitization, which involves allodynia and hyperalgesia, can be implemented using the threshold‐shift property of memristors, while adaptation and maladaptation can be implemented based on the adaptation of the memristors according to the applied pulses. Based on the adaptation rate, the sensory receptors can be classified into adaptive and maladaptive receptors. For sensory neurons, the characteristic of spike generation is critical, which process and transmit electrical signals (action potentials) to other neurons or associated organs. This can be implemented using various neuron models, especially the LIF model, which is a circuit constructed using threshold‐switching devices and manual component to simulate the behaviour of biological neurons. Low energy consumption characteristics are dependent on low discharging current and threshold voltage, as well as high on‐state resistance, while high‐speed computing relies on fast discharging speed accompanied by low off‐state resistance. Artificial sensory synapses, which are connections between neurons, are fundamental to neural communication, with synaptic plasticity playing a key role in learning and memory functions. These synapses modulate the input signal based on the synaptic weight, enabling synaptic plasticity, a fundamental process underlying learning and memory in the human brain. To emulate synapses, memristors must have non‐volatile properties to memorize responses to stimuli, and conductance modulation and linearity play an important role in implementing higher intelligence. Non‐volatile memristors must satisfy specific criteria such as variation, retention, endurance, on/off ratio, multilevel state, and linearity of conductance modulation, which are crucial for accurately reproducing synaptic functionalities in artificial system.

Then, we discuss the role of memristive devices in artificial sensory systems and their parallels with the human sensory system. We explore how a memristor‐based artificial sensory system detects external stimuli, exploiting the memristor‐based sensory elemental devices introduced in the previous sections. The artificial sensory system, implemented as a memristive device, consists of the sensory receptor system that detects the external stimuli and generates continuous receptor signals and the sensory neuron system that outputs the generated receptor signals by converting them into spike‐based signals such as action potential. In particular, the receptor system consists of a sensor unit that detects analog stimuli and converted them into electrical signals and a receptor device unit that processes the signals received from the sensor according to sensory receptor characteristics. Therefore, this review introduces the study of sensory receptor systems, divided into sensor‐integrated and single‐device receptor systems. We also explore the role of the memristor‐based neural networks, consisting of neurons and synapses, in mimicking brain function. In this process, a spike signal is generated via an artificial neuron based on memristors, mirroring the biological functionality in response to the sensed stimulus. These signals are then integrated and processed at the memristor‐based synapse through conductance modulation (as the role of synaptic weight). Drawing parallels to human synaptic plasticity, memristor synapses play a critical role in reinforcement learning, decision‐making processes, and perception. Each section previews discussions on artificial neural networks, pattern recognition, and classification problems with the memristor‐based artificial neural system.

So far, the practical application of memristor‐based artificial sensory systems is still in its infancy, and many challenges exist. For example, most studies have primarily focused on fabricating and improving individual sensory elements, and integrated artificial sensory systems with all functional elements have not yet been explored. Some studies have demonstrated partial integration of elements, but they are limited in terms of generality. Most studies are applicable only for a narrow range of specific stimuli, and this lack of generalization across a wide range of stimuli prevents the application and utilization of these systems in diverse applications. For functional integration between each sensory element device, it is important that the range of the output signal from the front module must be a value that the back module can receive and process. In particular, in order to develop a multimodal sensory system that can handle various stimuli, an accurate input and output signal range for the operating system is required, and the development of a sensor device compatible with the signal range is required accordingly. Moreover, the fabrication of memristive devices has its own set of challenges, including controlling the consistency of the memristive materials, optimizing device architecture, and addressing issues related to device endurance and retention. In particular, as the complexity of the systems increases, it becomes increasingly difficult to scale up while maintaining efficient operation, and the energy efficiency of these systems often falls short of that of biological sensory systems when expanding to include more sensors or operating at higher speeds. Therefore, an essential focus in the future development of memristor‐based artificial sensory systems should be on the development of integrated sensory systems that fabricate and integrate each sensory element in a reliable, scalable, and energy‐efficient manner. These advances require thorough investigation of the individual sensory elements, to achieve the desired functionality and efficiency, and this review provides an understanding of each element device and can be used as a guideline for the development of artificial sensory systems.

## CONFLICT OF INTEREST STATEMENT

The authors declare no conflicts of interest.
